# Synthesis of icariin from kaempferol through regioselective methylation and *para*-Claisen–Cope rearrangement

**DOI:** 10.3762/bjoc.11.135

**Published:** 2015-07-20

**Authors:** Qinggang Mei, Chun Wang, Zhigang Zhao, Weicheng Yuan, Guolin Zhang

**Affiliations:** 1 Chengdu Institute of Biology, Chinese Academy of Sciences, Chengdu 610041, China; 2 Chengdu Institute of Organic Chemistry, Chinese Academy of Sciences, Chengdu 610041, China; 3 College of Chemistry and Environmental Protection Engineering, Southwest University for Nationalities, Chengdu 610041, China

**Keywords:** Claisen–Cope rearrangement, flavonol, icariin, prenylation, regioselectivity

## Abstract

The hemisynthesis of the naturally occurring bioactive flavonoid glycoside icariin (**1**) has been accomplished in eleven steps with 7% overall yield from kaempferol. The 4′-OH methylation of kaempferol, the 8-prenylation of 3-*O*-methoxymethyl-4′-*O*-methyl-5-*O*-prenyl-7-*O*-benzylkaempferol (**8**) via *para*-Claisen–Cope rearrangement catalyzed by Eu(fod)_3_ in the presence of NaHCO_3_, and the glycosylation of icaritin (**3**) are the key steps.

## Introduction

**Figure 1:**
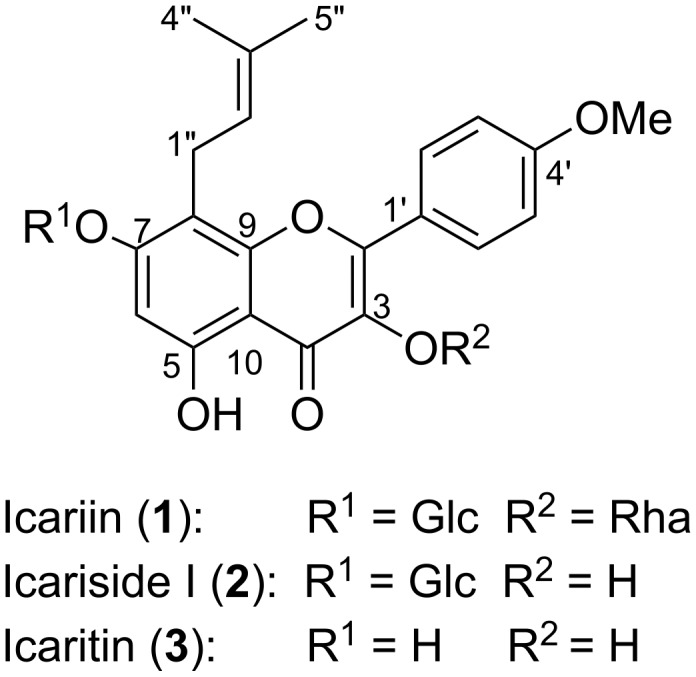
Structures of icariin (**1**), icariside I (**2**) and icaritin (**3**).

The plants of the Genus *Epimedium*, also known as “Yin-Yang-Huo”, were used in traditional Chinese medicine and are believed to invigorate the kidney and enhance the “Yang”. For more than one thousand years, some plants of *Epimedium* have been widely used in China to treat cardiovascular diseases, amnesia, arthritis, asthenia, impotence, infertility, lumbago and other chronic illnesses [1–2]. Icariin (**1**), a 3,7-diglycosylflavone (Figure 1), is recognized as the major pharmacologically active ingredient of these plants [3–4], and has been used as the index for quality control of the herbs and relative drug preparations [5]. Icariin, icariside I (**2**), and their aglycone, icaritin (**3**) (Figure 1), possess multiple biological activities, such as anti-oxidative [6], anti-inflammatory [7], anti-osteoporotic [8], anticancer [9], neuroprotective [10], angiogenesis stimulating [11], testosterone mimetic [12], antidepressant-like [13] and multidrug resistance reversal [14] activities.

Although icariin exerts a variety of important bioactivities, reports concerning its synthesis are very scarce. One Chinese patent reported a 15-step total synthesis of icariin from benzyl alcohol with 0.6% overall yield [15]. The 11-step synthesis of icaritin, starting from 2,4,6-trihydroxyacetophenone via microwave-assisted Claisen rearrangement reaction as the key step, was succeeded with an overall yield of 23% [16]. In view of the long synthetic routes, tedious work-up and harsh reaction conditions, an alternative access to icariin (**1**) and related compounds is demanded for large-scale production and practical application.

Our interest in flavonoids with activities such as estrogen biosynthesis regulation and PDE inhibition, prompted us to seek for a practically synthetic approach to icariin. Previously our group prepared icaritin from phloroglucin through an Algar–Flynn–Oyamada reaction and europium-promoted prenylation [17]. However, a number of challengable problems such as strict conditions, numerous byproducts and poor yields, were calling for our continuous efforts to overcome these hitches [18]. As a continuation of this program, herein we report a new approach to icaritin and then icariin through semi-synthesis from the commercially available natural product kaempferol. Our previously developed regioselective methylation of kaempferol [19], Europium(III)-catalyzed *para*-Claisen–Cope rearrangement and the bis-glycosylation are the key features of this linear synthesis. Previously, we succeeded in the selective methylation of 4′-OH in kaempferol. In this work, we focus on developing an efficient procedure for the selective prenylation of flavonols for facile access to icariin (**1**).

## Results and Discussion

**Scheme 1:**
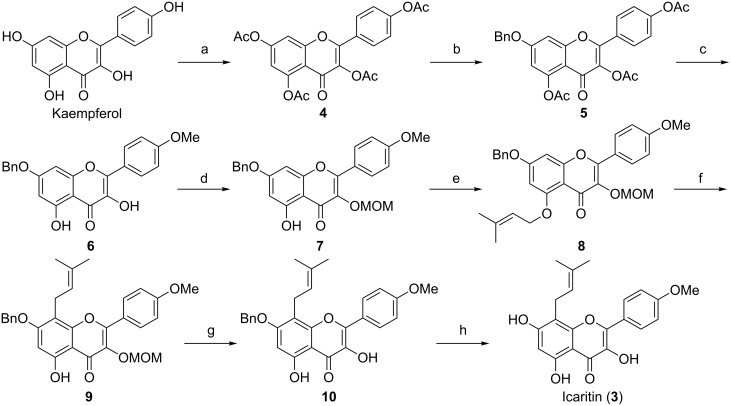
Reagents and conditions: (a) Ac_2_O, pyridine, 94%; (b) BnBr, KI, K_2_CO_3_, acetone, 85%; (c) Me_2_SO_4_, K_2_CO_3_, acetone, MeOH, 82%; (d) MOMCl, *N*,*N*-diisopropylethylamine (DIPEA), CH_2_Cl_2_, 93%; (e) 3,3-dimethylallyl bromide, 18-crown-6, K_2_CO_3_, acetone, 86%; (f) Eu(fod)_3_, NaHCO_3_, PhCl, 85 °C, 61%; (g) MeOH, 3 M HCl (aq), reflux, 95%; (h) Pd/C, 1,4-cyclohexadiene, MeOH, 84%.

Our synthetic approach to **1** commenced with the preparation of aglycone **3**, as illustrated in Scheme 1. First, 7-*O*-benzylkaempferide (**6**) was easily obtained from kaempferol through tetraacetylation, followed by benzylation at C-7 and selectively methylation, according to our previously developed procedure [19]. In order to methylate exclusively the 4′-OH in kaempferol, we initially attempted to use methoxymethyl (MOM) as 7-OH protecting group, but this method could not provide an ideal yield in the 4′-OH selective methylation and in subsequent Claisen–Cope rearrangement.

**Scheme 2:**
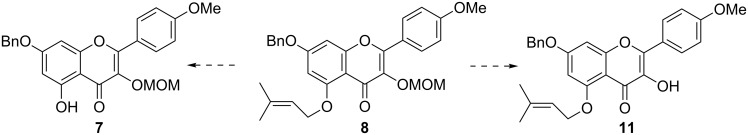
Decomposition of **8**.

The resulting free 3-OH in **6** is protected with an orthogonal protecting group to give 3-*O*-methoxymethyl-4′*-O*-methyl-7-*O*-benzylkaempferol (**7**) in 93% yield. Then introduction of a requisite prenyl ether moiety at C-5 of **7**, with 3,3-dimethylallyl bromide in the presence of potassium carbonate and 18-crown-6, formed **8**, whose all hydroxy groups were protected for the rearrangement accompanied in the next step. It is noticeable that this compound is slightly less stable in solution, easily decomposing into **7** and **11** (Scheme 2), especially under acidic conditions.

**Scheme 3:**
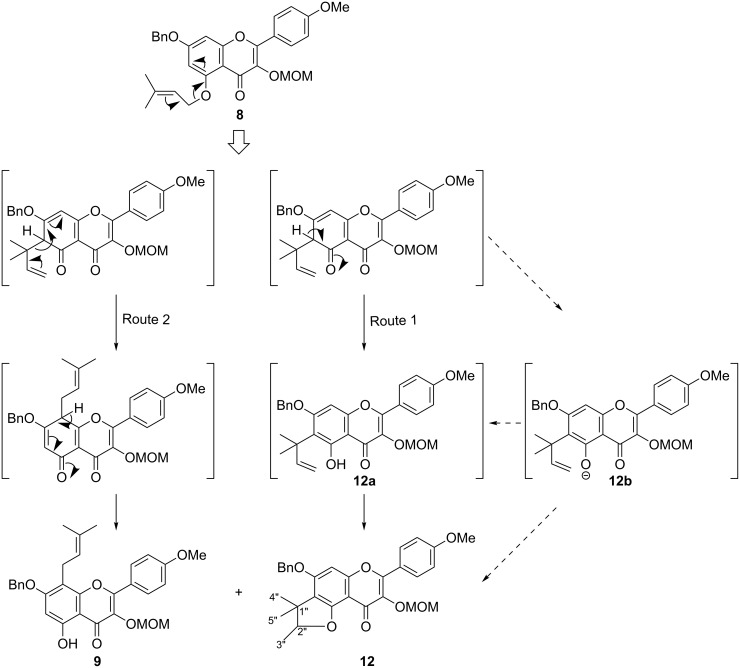
Claisen rearrangement of flavonol **8**.

The Claisen rearrangement is commonly accepted as an efficient method for the preparation of *C*-isopentenyl. In the case of flavonoids, control of the regioselectivity of *ortho* (C6*)*/*para* (C8*)-*rearranged products is still remained a challenging issue [20]. Once the prenyl ether **8** was obtained, attention was fastened on the *para*-Claisen–Cope rearrangement to acquire the 8-prenylated product. Two pathways have been developed for the rearrangement: direct heating at about 217 °C [17,21] and heating at about 60 °C in the presence of Eu(fod)_3_ [22–23]. When we adapted the former path, reflux of **8** in *N*,*N*-diethylaniline for 3 h led to complete consumption of **8** and gave an intractable product mixture, probably due to the prevalence of the 5-*O*-prenyl chain elimination with no rearrangement. Then we turned to try the latter method with 10 mol % Eu(fod)_3_ as the catalyst at 60 °C in dry CHCl_3_ (Table 1), in which the Claisen rearrangement of **8** indeed took place. Unlike reported results [24–26], we obtained the *ortho*-rearranged product, which was not the 6-(1,1-dimethylallyl) product **12a**, but an inseparable epimeric mixture of the dihydrofuro-flavonol **12**, presumably resulted from the cyclization of the 6-(1,1-dimethylallyl) chain with the 5-hydroxy group (Scheme 3). The ratio of the *para*-rearranged product **9** to **12** was 0.7:1. When PhCl was used as solvent, the rearrangement of **8** at 85 ºC for 24 h allowed the isolation of **9** and **12** in a 0.9:1 ratio (Table 1). Although the proportion of **9** and the total yield were improved, the *para/ortho-*selectivity was still unsatisfactory, even when prolonging the reaction time to 36 h or raising the temperature to 100 °C. By carefully investigating the two routes in Scheme 3, we assumed that an appropriate base is in favor of the [1,5]H σ migration in route 2, as well as lowering the acidity of Eu(fod)_3_ to prevent **12b** from cyclization in route 1, and therefore enhances the proportion of the *para-*rearrangement product **8**. Obviously, strong alkali could lead to heterocycle cleavage of the flavonoid skeleton. Gratifyingly, when prenyl ether **8** was treated with catalytic amounts of Eu(fod)_3_ and NaHCO_3_ in PhCl at 85 °C for 24 h (Table 1), the Claisen *para*-rearranged product was obtained selectively in 61% yield with the ratio **9** to **12** of 2.1:1. With other bases such as K_2_CO_3_ or DIPEA, the results were very different, probably due to the partial inactivity of Eu(fod)_3_ under these conditions. It is noteworthy that NaHCO_3_ only affected the **9**/**12** ratio, but not the total amount of *C*-isoprenoid flavonols **9** and **12**, which was found to stay around 90%. This to some extent, implies that our hypothesis on using appropriate base to hinder the Route 1 and to promote the Cope rearrangement step from C-6 to C-8 could be true. Therefore, the combination of Eu(fod)_3_ and NaHCO_3_ is preferable for the Claisen–Cope rearrangement from 5-*O*- to 8-*C*-prenyl products.

**Table 1:** Rearrangement of 5-*O*-prenylflavone **8**.

Method^a^	*T* (ºC)	*t* (h)	Products (%)^b^	Ratio **9**:**12**	Total **9** + **12**

CHCl_3_, Eu(fod)_3_	60	24	**9** (33), **12** (46)	0.7:1	79%
PhCl, Eu(fod)_3_	85	24	**9** (42), **12** (47)	0.9:1	89%
PhCl, Eu(fod)_3_, NaHCO_3_	85	24	**9** (61), **12** (29)	2.1:1	90%

^a^Eu(fod)_3_: 10 mol %; NaHCO_3_: 100 mol %. ^b^Isolated yield.

Exposure of compound **9** to dilute methanolic HCl at reflux resulted in the cleavage of the methoxymethyl group, giving rise to 7-*O*-benzylicaritin (**10**). Removal of the benzyl group in **10** resulting in **3** without affecting the double bond of the prenyl residue was another challenge. The hydrogenation of **10** with H_2_–Pd/C using EtOAc/MeOH (1:1) as solvent at 10 °C for 3 h, followed by column chromatography and recrystallization, provided **3** in 39% yield along with a large amount of the byproduct with reduced double bond. Then we tried a transfer hydrogenolysis method using ammonium formate/10% Pd/C [27], but the result was not improved. The desired product icaritin (**3**) was finally obtained in 84% yield from **10** when we used 1,4-cyclohexadiene/10% Pd/C in MeOH [28]. Hence, we optimized a convenient eight-step sequence from kaempferol to **3** through regioselective methylation and a domino Claisen–Cope rearrangement that delivered **3** in 26% overall yield. The NMR spectra of synthetic **3** (see Supporting Information File 1 for experimental and spectral data) were in accordance with the reported ones [16,29].

**Scheme 4:**
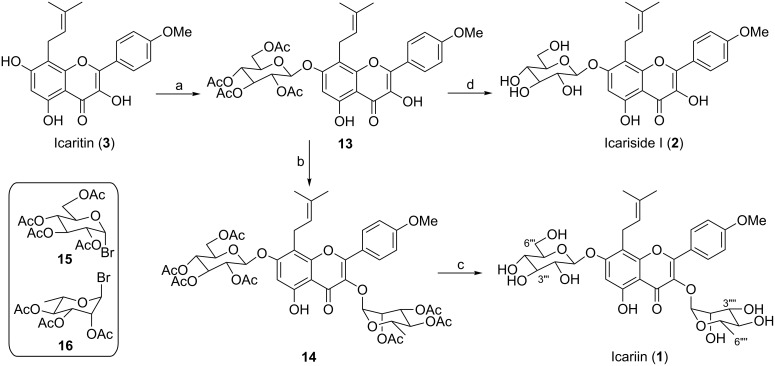
Reagents and conditions: (a) **15**, DMF/CHCl_3_, Ag_2_CO_3_, molecular sieves (4 Å, powder); (b) **16**, CH_2_Cl_2_, Ag_2_O, molecular sieves (4 Å powder), 31% for 2 steps; (c) NH_3_ (g), MeOH, 94%; (d) NH_3_ (g), MeOH, 63% for 2 steps.

With icaritin (**3**) in hand, the selective glycosylation was investigated (Scheme 4). The alkylation of OH in kaempferol followed a specific reactivity order: 7 > 4′ > 3 >> 5 [19]. We initially attempted the 7-OH glycosylation with tetra-*O*-acetylglucopyranosyl bromide (**15**) [30] as the donor and SrCO_3_ or AgNO_3_ as an activator in pyridine or quinoline [15,31], but it was unsuccessful. TLC monitoring revealed that most of icartin was still unreacted even prolonging the reaction time to 48 h. To our delight, switching the solvent to DMF/CHCl_3_ and the activator to Ag_2_CO_3_ made the reaction to proceed smoothly, resulting in the synthesis of **13**. The yellow powder **13** was treated with tri-*O*-acetylrhamnopyranosyl bromide (**16**) [30] in CH_2_Cl_2_ using Ag_2_O as activator, following a known procedure [32]. Compound **14** was easily separated by normal silica gel column chromatography, with a yield of 31% from **3**.

The final procedure in completing the synthesis of icariin (**1**) is the removal of all acetyl groups in **14** (Scheme 4). In contrast to the traditional method for deacetylation under basic conditions such as with CH_3_ONa [15] or K_2_CO_3_ [33] in CH_3_OH, employing methanolic ammonia (7.0 M) for 3 h at room temperature effectively suppressed the cleavage of the sugar moiety [34] and gave the target compound **1** in high conversion. Icariside I (**2**) was also obtained using the same procedure from compound **13**. Thus, icariin (**1**) and icariside I (**2**) were synthesized from kaempferol with 7% and 16% overall yields, respectively.

The compounds **14**, **2** and **1** were fully characterized by ESI-HRMS, ^1^H NMR, ^13^C NMR, ^1^H,^1^H COSY, ^1^H,^13^C HMBC/HSQC and IR (see Supporting Information File 1 for experimental and spectral data). In icariin (**1**), the configurations of the anomeric C-atoms of rhamnose and glocuse were α and β on the basis of the anomeric proton signals at δ 5.27 and 5.00 with *J* = 1.3 Hz and 7.4 Hz, respectively. The NMR data of icariin (**1**) were in agreement with those reported [29,35] and with the spectra of the authentic, naturally derived sample.

## Conclusion

In conclusion, we have developed an efficient and practical procedure for the preparation of the pharmacologically important compounds icariin (**1**) and icariside I (**2**) from kaempferol with 7% and 16% overall yields, respectively. The addition of NaHCO_3_ to the Eu(fod)_3_-catalyzed Claisen–Cope rearrangement could inhibit the formation of the *ortho*-cyclized rearrangement product, thus selectively promoting the *para*-Claisen–Cope rearrangement of flavonoid 5-prenyl ether*.* This procedure could afford facile access to the derivatives of icariin (**1**) and other structurally related flavonoid analogues.

## Supporting Information

File 1 Experimental section and copies of NMR, ESI-HRMS, HMBC, HSQC, COSY and NOESY spectra.
