# Supplementary material for: Synthesis of icariin from kaempferol through regioselective methylation and para-Claisen–Cope rearrangement
Source: Beilstein J Org Chem. 2015 Jul 20;11:1220–5. doi: 10.3762/bjoc.11.135 (PMC4578360; doi:10.3762/bjoc.11.135)

Supporting Information
for
Synthesis of icariin from kaempferol through regioselective methylation and *para*-Claisen–Cope rearrangement

Qinggang Mei[1,2], Chun Wang[1], Zhigang Zhao[3], Weicheng Yuan[2] and Guolin Zhang*[1]

Address: [1]Chengdu Institute of Biology, Chinese Academy of Sciences, Chengdu 610041, China, [2]Chengdu Institute of Organic Chemistry, Chinese Academy of Sciences, Chengdu 610041, China and [3]College of Chemistry and Environmental Protection Engineering, Southwest University for Nationalities, Chengdu 610041, China

Email: Guolin Zhang* - zhanggl@cib.ac.cn

*Corresponding author

Experimental section and copies of NMR, ESI-HRMS, HMBC, HSQC, COSY and NOESY spectra

Contents	Page No.
Experimental section	S2–S11
^{1}H NMR spectra of **1, 2, 3, 7, 8, 9, 10, 12, 14**	S12–S20
^{13}C NMR spectra of **1, 2, 3, 7, 8, 9, 10, 12, 14**	S21–S29
ESI-HRMS spectra of **1, 2, 3, 7, 8, 9, 10, 12, 14**	S30–S38
HMBC spectra of **1, 2, 3, 7 ,8, 9, 12, 14**	S39–S46
HSQC/COSY spectra of **1, 2, 14;** NOESY Spectrum of **3**	S47–S53

Experimental Section

Melting points were determined by an X-6 apparatus without correction. Optical rotations were measured with a Perkin-Elmer M341 automatic polarimeter. IR spectra were recorded on a Perkin-Elmer Spectrum One spectrometer (KBr disc) and NMR spectra on a Bruker AC-400 spectrometer (^{1}H: 400 MHz; ^{13}C: 100 MHz) with DMSO-d_6 (δ 2.50/39.52) as solvent and internal standard at ambient temperature. The 2D NMR spectra (COSY, NOESY and ^{1}H-^{13}C HMBC, HSQC) were conducted using standard software. The ESI-HRMS was carried out on a Bruker Bio TOF IIIQ (quadrupole time of flight) mass spectrometer. Preparative TLC was performed using commercially available precoated glass silica gel GF_{254} plates of 0.15–0.2 mm thickness. Column chromatography was carried out with 200–300 mesh silica gel using the flash technique.

Unless otherwise specified, chemicals and solvents were of analytical reagent grade and used as obtained from commercial sources without further purification. Kaempferol was from Yangling Dongke Maidisen Pharmaceutical Co., Ltd., China. Icariin reference substance was purchased from Aladdin Reagent (Shanghai) Co., Ltd. DMF was dried with 4 Å molecular sieves and distilled at reduced pressure. PhCl was freshly distilled from sodium benzophenone ketyl prior to use. CH_2Cl_2 and $CHCl_3$ were freshly distilled over CaH_2 and acetone was freshly distilled from potassium carbonate prior to use.

Kaempferol was converted to 7-*O*-benzylkaempferide (**6**) following the reported procedure [1]. 2,3,4,6-Tetra-*O*-acetyl-α-D-glucopyranosyl bromide (**15**) and 2,3,4-tri-*O*-acetyl-α-L-rhamnopyranosyl bromide (**16**) were synthesized according to literature proecdures [2].

3-*O*-Methoxymethyl-4′-*O*-methyl-7-*O*-benzylkaempferol (7)

To a solution of 7-*O*-benzylkaempferide (**6**, 3.12 g, 8 mmol) and iPr_2NEt (3.76 mL, 21.6 mmol) in CH_2Cl_2 (80 mL), chloromethyl methyl ether (0.85 mL, 11.2 mmol) was added at 0 °C. The mixture was stirred for 6 h at room temperature, and was acidified cautiously with 0.5 M HCl (aq) until pH = 5. The organic phase was washed with brine (50 mL), dried over $MgSO_4$, and evaporated under reduced pressure. The dried residue was recrystallized from EtOAc/95% ethanol (1:5) to provide **7** as yellow needles. Yield: 3.24 g (93%); mp: 116–117 ºC. IR (cm^{-1}): 3444, 2919, 1664, 1607, 1497, 1351, 1306, 1265, 1221, 1174, 1082, 1025. ^{1}H NMR (400 MHz, DMSO-d_6) δ 12.55 (s, 1H, OH-5), 8.05 (d, *J* = 8.9 Hz, 2H, H-2′/6′), 7.47 (d, *J* = 7.3 Hz, 2H, H-2‴/6‴), 7.41 (t, *J* = 7.3 Hz, 2H, H-3‴/5‴), 7.36 (d, *J* = 7.3 Hz, 1H, H-4‴), 7.15 (d, *J* = 8.9 Hz, 2H, H-3′/5′), 6.85 (d, *J* = 2.0 Hz, 1H, H-8), 6.48 (d, *J* = 2.0 Hz, 1H, H-6), 5.24 (s, 2H, H-7‴), 5.14 (s, 2H, OCH_2O), 3.86 (s, 3H, OCH_3-4′), 3.14 (s, 3H, CH_2OCH_3). ^{13}C NMR (100 MHz, DMSO-d_6) δ 177.83 (C-4), 164.22 (C-7), 161.40 (C-4′), 160.94 (C-5), 156.40 (C-2), 156.34 (C-9), 136.06 (C-1‴), 134.72 (C-3), 130.46 (C-2′/6′), 128.50 (C-3‴/5‴), 128.10 (C-4‴), 127.81 (C-2‴/6‴), 122.08 (C-1′), 114.06 (C-3′/5′), 105.24 (C-10), 98.52 (C-6), 97.19 (OCH_2O), 93.30 (C-8), 70.01 (C-7‴), 57.04 (CH_2OCH_3), 55.44 (OCH_3-4′). ESI-HRMS *m*/*z*: 457.1258 $[M+Na]^+$ (calcd for $C_{25}H_{22}O_7Na$: 457.1258).

3-*O*-Methoxymethyl-4′-*O*-methyl-5-*O*-isopentenyl-7-*O*-benzylkaempferol (8)

To a well stirred suspension of **7** (2.5 g, 5.76 mmol), 18-crown-6 (0.05 g, 0.19 mmol) and anhydrous potassium carbonate (2.07 g, 15 mmol) in dry acetone (70 mL), a solution of 3,3-dimethylallyl bromide (1.94 mL, 16.13 mmol) in dry acetone (8 mL) was added dropwise over 20 min at room temperature. The resulting suspension was continuously stirred for 19 h. After evaporation of the filtrate under reduced pressure,

the obtained yellow viscous oil was dissolved in CH_2Cl_2 and subjected to silica gel column chromatography with EtOAc/petroleum ether (1:3) as an eluent to offer an off-white solid. Recrystallization from CH_2Cl_2-95% ethanol (1:9) provided the desired compound **8** as off-white crystals. Yield: 2.49 g (86%); mp: 79–81 ºC. IR (cm^{-1}): 2935, 1633, 1608, 1575, 1455, 1350, 1295, 1262, 1180, 1099, 1035, 826. ^{1}H NMR (400 MHz, DMSO-d_6) δ 8.02 (d, *J* = 8.9 Hz, 2H, H-2′/6′), 7.49 (d, *J* = 7.4 Hz, 2H, H-2‴/6‴), 7.42 (t, *J* = 7.4 Hz, 2H, H-3‴/5‴), 7.37 (t, *J* = 7.4 Hz, 1H, H-4‴), 7.12 (d, *J* = 8.9 Hz, 2H, H-3′/5′), 6.88 (d, *J* = 2.1 Hz, 1H, H-8), 6.60 (d, *J* = 2.1 Hz, 1H, H-6), 5.47 (brs, 1H, H-2″), 5.24 (s, 2H, H-7‴), 5.09 (s, 2H, OCH_2O), 4.62 (d, *J* = 6.5 Hz, 2H, H-1″), 3.85 (s, 3H, OCH_3-4′), 3.11 (s, 3H, CH_2OCH_3), 1.76 (s, 3H, H-5″), 1.72 (s, 3H, H-4″). ^{13}C NMR (100 MHz, DMSO-d_6) δ 171.94 (C-4), 162.61 (C-7), 160.83 (C-4′), 159.50 (C-5), 158.16 (C-9), 152.40 (C-2), 136.86 (C-3″), 136.75 (C-3), 136.15 (C-1‴), 129.99 (C-2′/6′), 128.50 (C-3‴/5‴), 128.12 (C-4‴), 127.93 (C-2‴/6‴), 122.53 (C-1′), 119.57 (C-2″), 113.92 (C-3′/5′), 108.67 (C-10), 97.55 (C-6), 96.68 (O*C*H_2O), 93.83 (C-8), 69.95 (C-7‴), 65.86 (C-1″), 56.88 (CH_2O*C*H_3), 55.36 (O*C*H_3-4′), 25.44 (C-5″), 18.10 (C-4″). ESI-HRMS *m*/*z*: 503.2075 $[M+H]^+$ (calcd for $C_{30}H_{31}O_7$: 503.2064).

3-*O*-Methoxymethyl-7-*O*-benzylicaritin (9) and 4-benzyloxy-2,3,3-trimethyl-7-(4-methoxyphenyl)-8-methoxymethoxy-2,3-dihydrofuro[2,3-*f*]chromen-9-one (12)

A solution of **8** (3 g, 5.98 mmol) in dry PhCl (80 mL) was mixed with $Eu(fod)_3$ (0.62 g, 0.6 mmol) and $NaHCO_3$ (0.5 g, 6 mmol) under nitrogen atmosphere. After stirring for 24 h at 85 ºC, the mixture was cooled, filtered and evaporated to dryness. The dried residue was subjected to silica gel column chromatography eluting with petroleum ether/CH_2Cl_2/EtOAc (6:3:0.5). 8-Prenyl derivative **9** was obtained after evaporation of solvents. A diastereomeric mixture of dihydrofuro[2,3-*f*]chromone **12** was succeeded by further elution with acetone/petroleum ether (1:3).

Compound **9**: pale yellow powder, yield 1.84 g (61%), mp 151–153 ºC. IR (cm^{-1}): 3438, 2924, 2853, 1652, 1610, 1594, 1438, 1377, 1301, 1253, 1178, 1087, 840. ^{1}H NMR (400 MHz, DMSO-d_6) δ 12.61 (s, 1H, OH-5), 8.02 (d, *J* = 9.0 Hz, 2H, H-2′/6′), 7.46 (d, *J* = 7.1 Hz, 2H, H-2‴/6‴), 7.41 (t, *J* = 7.1 Hz, 2H, H-3‴/5‴), 7.35 (t, *J* = 7.1 Hz, 1H, H-4‴), 7.15 (d, *J* = 9.0 Hz, 2H, H-3′/5′), 6.66 (s, 1H, H-6), 5.26 (s, 2H, H-7‴), 5.15 (brs, 1H, H-2″), 5.12 (s, 2H, OCH_2O), 3.85 (s, 3H, OCH_3-4′), 3.46 (d, *J* = 6.6 Hz, 2H, H-1″), 3.11 (s, 3H, CH_2OCH_3), 1.60 (s, 6H, H-4″/5″). ^{13}C NMR (100 MHz, DMSO-d_6) δ 178.18 (C-4), 161.48 (C-7), 161.42 (C-4′), 159.45 (C-5), 156.44 (C-2), 152.87 (C-9), 136.34 (C-1‴), 134.34 (C-3), 131.41 (C-3″), 130.40 (C-2′/6′), 128.49 (C-3‴/5‴), 128.05 (C-4‴), 127.62 (C-2‴/6‴), 122.02 (C-1′), 121.92 (C-2″), 114.15 (C-3′/5′), 107.43 (C-8), 104.71 (C-10), 97.20 (OCH_2O), 96.33 (C-6), 70.20 (C-7‴), 57.04 (CH_2OCH_3), 55.47 (OCH_3-4′), 25.41 (C-5″), 21.35 (C-1″), 17.72 (C-4″). ESI-HRMS *m*/*z*: 503.2079 $[M+H]^+$ (calcd for $C_{30}H_{31}O_7$: 503.2064).

Compound **12**: pale yellow glassy solid, yield 0.87 g (29%), mp 72–74 ºC. IR (cm^{-1}): 2960, 2929, 1626, 1607, 1445, 1347, 1298, 1257, 1183, 1071, 943. ^{1}H NMR (400 MHz, DMSO-d_6) δ 8.00 (d, *J* = 9.0 Hz, 2H, H-2′/6′), 7.49 (d, *J* = 7.2 Hz, 2H, H-2‴/6‴), 7.44 (t, *J* = 7.2 Hz, 2H, H-3‴/5‴), 7.36 (t, *J* = 7.2 Hz, 1H, H-4‴), 7.12 (d, *J* = 9.0 Hz, 2H, H-3′/5′), 6.90 (s, 1H, H-8), 5.27 (s, 2H, H-7‴), 5.09 (s, 2H, OCH_2O), 4.52 (q, *J* = 6.6 Hz, 1H, H-2″), 3.85 (s, 3H, OCH_3-4′), 3.09 (s, 3H, CH_2OCH_3), 1.38 (s, H-4″ or 5″), 1.37 (d, *J* = 6.6 Hz, 3H, H-3″), 1.12 (s, 3H, H-4″ or 5″). ^{13}C NMR (100 MHz, DMSO-d_6) δ 171.80 (C-4), 160.91 (C-4′), 158.89 (C-7), 157.55 (C-9), 156.76 (C-5), 153.67 (C-2), 136.37 (C-3), 136.31 (C-1‴), 130.10 (C-2′/6′), 128.59 (C-3‴/5‴), 128.06 (C-4‴), 127.44 (C-2‴/6‴), 122.63 (C-1′), 119.50 (C-6), 113.92 (C-3′/5′), 105.44 (C-10), 96.69 (OCH_2O), 93.49 (C-8), 90.24 (C-2″), 69.94 (C-7‴), 56.88 (CH_2OCH_3), 55.39 (OCH_3-4′), 42.94 (C-1″), 25.15 (C-4″ or 5″), 20.71 (C-4″ or 5″), 13.96 (C-3″). ESI-HRMS *m*/*z*:

525.1900 [M+Na]$^+$ (calcd for $C_{30}H_{30}O_7Na$: 525.1884).

7-*O*-Benzylicaritin (10)

To a solution of **9** (2.01 g, 4 mmol) in MeOH (120 mL), 3 M HCl (aq) (30 mL) was added under nitrogen atmosphere. The reaction mixture was refluxed for 2.5 h, and then half of the solvent was removed under reduced pressure. The residue was neutralized with saturated $NaHCO_3$ solution and retained for 12 h at room temperature. Abundant yellow precipitates were recovered by filtration. After washing with ethanol, the crude product was recrystallized from petroleum ether/EtOAc (4:1) to give **10** as yellow granules. Yield: 1.74 g (95%); mp: 185–187 ºC. IR (cm^{-1}): 3304, 2928, 1648, 1616, 1590, 1552, 1511, 1317, 1261, 1171, 1076, 985. ^{1}H NMR (400 MHz, DMSO-d_6) δ 12.51 (s, 1H, OH-5), 9.61 (s, 1H, OH-3), 8.14 (d, J = 9.1 Hz, 2H, H-2′/6′), 7.46 (d, J = 6.9 Hz, 2H, H-2‴/6‴), 7.42 (t, J = 7.3 Hz, 2H, H-3‴/5‴), 7.35 (t, J = 7.1 Hz, 1H, H-4‴), 7.14 (d, J = 9.1 Hz, 2H, H-3′/5′), 6.64 (s, 1H, H-6), 5.26 (s, 2H, H-7‴), 5.15 (t, J = 6.9 Hz, 1H, H-2″), 3.84 (s, 3H, OCH_3-4′), 3.49 (d, J = 6.9 Hz, 2H, H-1″), 1.63 (s, 3H, H-4″), 1.61 (s, 3H, H-5″). ^{13}C NMR (100 MHz, DMSO-d_6) δ 176.53 (C-4), 161.20 (C-7), 160.66 (C-4′), 158.99 (C-5), 152.67 (C-9), 146.81 (C-2), 136.46 (C-1‴), 136.03 (C-3), 131.39 (C-3″), 129.34 (C-2′/6′), 128.52 (C-3‴/5‴), 128.07 (C-4‴), 127.65 (C-2‴/6‴), 123.48 (C-1′), 122.10 (C-2″), 114.18 (C-3′/5′), 107.21 (C-8), 103.73 (C-10), 95.79 (C-6), 70.20 (C-7‴), 55.44 (OCH_3-4′), 25.46 (C-5″), 21.40 (C-1″), 17.77 (C-4″). ESI-HRMS *m*/*z*: 481.1620 [M+Na]$^+$ (calcd for $C_{28}H_{26}O_6Na$: 481.1622).

Icaritin (3)

A mixture of **10** (1.37 g, 3 mmol), 10% palladium on carbon (1.5 g) and 1,4-cyclohexadiene (2.9 mL, 30 mmol) in MeOH (110 mL) was stirred at room temperature for 2 h, followed by filtration through celite, rinsed with EtOAc (20 mL).

The filtrate was concentrated and recrystallized from MeOH to afford pure icaritin (**3)** as yellow needles. Yield: 0.93 g (84%); mp: 207–208 ºC; IR (cm^{-1}): 3319, 2927, 1626, 1603, 1536, 1422, 1379, 1318, 1257, 1178, 1149, 1036, 838; ^{1}H NMR (400 MHz, DMSO-d_6) δ 12.37 (s, 1H, OH-5), 10.74 (s, 1H, OH-7), 9.48 (s, 1H, OH-3), 8.12 (d, J = 9.1 Hz, 2H, H-2′/6′), 7.13 (d, J = 9.1 Hz, 2H, H-3′/5′), 6.29 (s, 1H, H-6), 5.17 (t, J = 6.9 Hz, 1H, H-2″), 3.84 (s, 3H, OCH_3-4′), 3.43 (d, J = 6.9 Hz, 2H, H-1″), 1.75 (s, 3H, H-4″), 1.63 (s, 3H, H-5″); ^{13}C NMR (100 MHz, DMSO-d_6) δ 176.22 (C-4), 161.22 (C-7), 160.47 (C-4′), 158.29 (C-5), 153.51 (C-9), 146.19 (C-2), 135.90 (C-3), 131.00 (C-3″), 129.16 (C-2′/6′), 123.55 (C-1′), 122.46 (C-2″), 114.09 (C-3′/5′), 105.62 (C-8), 103.06 (C-10), 97.81 (C-6), 55.38 (OCH_3-4′), 25.41 (C-5″), 21.18 (C-1″), 17.81 (C-4″); ESI-HRMS m/z: 369.1328 $[M+H]^+$ (calcd for $C_{21}H_{21}O_6$: 369.1333). The ^{1}H and ^{13}C NMR data of icaritin (**3**) were in agreement with those reported [3,4].

7-*O*-(2‴,3‴,4‴,6‴-Tetra-*O*-acetyl-β-D-glucopyranosyl)icaritin (13)

To a stirred suspension containing **3** (0.8 g, 2.17 mmol), Ag_2CO_3 (0.78 g, 2.83 mmol) and molecular sieves (4 Å powder) (1.2 g) in a mixed-solvent of DMF (2 mL) and $CHCl_3$ (12 mL), **15** (1.16 g, 2.83 mmol) in $CHCl_3$ (6 mL) was added. The mixture was stirred in dark at room temperature for 30 h under nitrogen, then diluted with 10 mL of $CHCl_3$ and filtered through celite eluting with $CHCl_3$. The filtrate was extracted with 0.3 M HCl (aq) (25 mL), washed with saturated $NaHCO_3$ solution, brine and dried over $MgSO_4$. After evaporation of solvents, **13** was collected as yellow powder (1.13 g), which was used in the next step without further purification.

3-*O*-(2⁗,3⁗,4⁗-Tri-*O*-acetyl-α-L-rhamnopyranosyl)-7-*O*-(2‴,3‴,4‴,6‴-tetra-*O*-acetyl-β-D-glucopyranosyl)icaritin (14)

A mixture of the above powder **13** (0.89 g), rhamnose bromide **16** (0.66 g, 1.88 mmol), and Ag_2O (0.44 g, 1.88 mmol) was stirred in the presence of molecular sieves (4 Å

powder) (1.5 g) in CH_2Cl_2 (20 mL) under nitrogen for 32 h, followed by filtration through celite and washed with CH_2Cl_2. The filtrate was concentrated under reduced pressure and the residue was purified over silica gel column chromatography with CH_2Cl_2/EtOAc (9:1). Compound **14** was collected as yellow needles. Yield: 514 mg (31%, for 2 steps); mp: 194–196 ºC; $[\alpha]_D^{20}$ –111.8 (*c* 0.17, $CHCl_3$). IR (cm^{-1}): 2925, 1756, 1652, 1599, 1434, 1372, 1222, 1180, 1045. ^{1}H NMR (400 MHz, DMSO-d_6) δ 12.41 (s, 1H, OH-5), 7.87 (d, *J* = 9.0 Hz, 2H, H-2′/6′), 7.15 (d, *J* = 9.0 Hz, 2H, H-3′/5′), 6.64 (s, 1H, H-6), 5.72 (d, *J* = 7.9 Hz, 1H, H-1‴), 5.53 (dd, *J* = 3.3, 1.6 Hz, 1H, H-2⁗), 5.47 (d, *J* = 1.6 Hz, 1H, H-1⁗), 5.42 (t, *J* = 9.6 Hz, 1H, H-3‴), 5.18 – 5.10 (m, 2H, H-2‴/3⁗), 5.02 (t, *J* = 9.6 Hz, 2H, H-2″/4‴), 4.79 (t, *J* = 10.0 Hz, 1H, H-4⁗), 4.41 – 4.33 (m, 1H, H-5‴), 4.21 (dd, *J* = 12.2, 6.4 Hz, 1H, H-6‴b), 4.09 (d, *J* = 10.2 Hz, 1H, H-6‴a), 3.85 (s, 3H, OCH_3-4′), 3.37 – 3.26 (m, 3H, H-1″/5⁗), 2.09 (s, 3H, CH_3COO), 2.04 (s, 3H, CH_3COO), 2.02 (s, 6H, 2×CH_3COO), 1.98 (s, 6H, 2×CH_3COO), 1.96 (s, 3H, CH_3COO), 1.62 (s, 3H, H-4″), 1.60 (s, 3H, H-5″), 0.77 (d, *J* = 6.2 Hz, 3H, H-6⁗). ^{13}C NMR (100 MHz, DMSO-d_6) δ 177.94 (C-4), 170.08 (CH_3*C*OO), 169.75 (CH_3*C*OO), 169.72 (CH_3*C*OO), 169.64 (CH_3*C*OO), 169.54 (CH_3*C*OO), 169.50 (CH_3*C*OO), 169.16 (CH_3*C*OO), 161.83 (C-4′), 159.15 (C-7), 159.12 (C-5), 157.99 (C-2), 153.28 (C-9), 134.02 (C-3), 131.73 (C-3″), 130.69 (C-2′/6′), 121.70 (C-1′), 121.59 (C-2″), 114.31 (C-3′/5′), 108.63 (C-8), 106.32 (C-10), 98.04 (C-6), 98.01 (C-1⁗), 96.80 (C-1‴), 71.83 (C-3‴), 71.15 (C-5‴), 70.56 (C-2‴), 69.37 (C-4⁗), 68.51 (C-2⁗), 68.26 (C-3⁗), 68.11 (C-4‴), 67.95 (C-5⁗), 61.78 (C-6‴), 55.67 (O*C*H_3-4′), 25.47 (C-5″), 21.23 (C-1″), 20.60 (*C*H_3COO), 20.51 (*C*H_3COO), 20.50 (2×*C*H_3COO), 20.46 (*C*H_3COO), 20.38 (2×*C*H_3COO), 17.85 (C-4″), 16.86 (C-6⁗). ESI-HRMS *m*/*z*: 971.3174 $[M+H]^+$ (calcd for $C_{47}H_{55}O_{22}$: 971.3179).

Icariin (1)

The solution of compound **14** (0.44 g, 0.45 mmol) in 7.0 M methanolic ammonia (15 mL) was stirred at room temperature for 3 h. The solution was concentrated and kept overnight at room temperature. Yellow powdery crystals of **1** were collected by filtration, washed with petroleum ether, and dried in vacuum. Yield: 286 mg (94%); mp: 224–226 ºC; $[\alpha]_D^{20}$ –120.0 (*c* 0.08, CH_3OH). IR (cm^{-1}): 3368, 2928, 1651, 1598, 1503, 1440, 1304, 1259, 1182, 1074. ^1H NMR (400 MHz, DMSO-d_6) δ 12.56 (s, 1H, OH-5), 7.89 (d, *J* = 9.0 Hz, 2H, H-2′/6′), 7.12 (d, *J* = 9.0 Hz, 2H, H-3′/5′), 6.62 (s, 1H, H-6), 5.41 (d, *J* = 4.7 Hz, 1H, OH-2‴), 5.27 (d, *J* = 1.3 Hz, 1H, H-1⁗), 5.18 (d, *J* = 4.3 Hz, 1H, OH-3‴), 5.14 (t, *J* = 7.6 Hz, 1H, H-2″), 5.11 (d, *J* = 5.4 Hz, 1H, OH-4‴), 5.04 (d, *J* = 4.6 Hz, 1H, OH-2⁗), 5.00 (d, *J* = 7.4 Hz, H-1‴), 4.79 (d, *J* = 4.9 Hz, 1H, OH-4⁗), 4.71 (d, *J* = 5.8 Hz, 1H, OH-3⁗), 4.68 (t, *J* = 5.5 Hz, 1H, OH-6‴), 3.99 (t, *J* = 4.6 Hz, 1H, H-2⁗), 3.85 (s, 3H, OC*H*$_3$-4′), 3.71 (dd, *J* = 10.1, 5.3 Hz, 1H, H-6‴b), 3.56 (dd, *J* = 14.6, 7.6 Hz, 1H, H-1″b), 3.51 – 3.47 (m, 1H, H-3⁗), 3.44 – 3.37 (m, 3H, H-1″a/5‴/6‴a, overlapped with H_2O), 3.31 – 3.27 (m, 2H, H-2‴/3‴), 3.19 – 3.02 (m, 3H, H-4‴/4⁗/5⁗), 1.68 (s, 3H, H-4″), 1.59 (s, 3H, H-5″), 0.77 (d, *J* = 6.0 Hz, 3H, H-6⁗); ^{13}C NMR (100 MHz, DMSO-d_6) δ 178.39 (C-4), 161.52 (C-4′), 160.60 (C-7), 159.18 (C-5), 157.49 (C-2), 153.12 (C-9), 134.72 (C-3), 131.30 (C-3″), 130.71 (C-2′/6′), 122.34 (C-1′), 122.21 (C-2″), 114.20 (C-3′/5′), 108.41 (C-8), 105.69 (C-10), 102.06 (C-1⁗), 100.59 (C-1‴), 98.21 (C-6), 77.27 (C-5‴), 76.67 (C-3‴), 73.45 (C-2‴), 71.18 (C-4⁗), 70.83 (C-5⁗), 70.39 (C-3⁗), 70.18 (C-2⁗), 69.74 (C-4‴), 60.71 (C-6‴), 55.63 (O*C*H_3-4′), 25.59 (C-5″), 21.53 (C-1″), 17.98 (C-4″), 17.56 (C-6⁗). ESI-HRMS *m*/*z*: 699.2256 $[M+Na]^+$ (calcd for $C_{33}H_{40}O_{15}Na$: 699.2259). The ^1H and ^{13}C NMR data of icariin (**1**) were in agreement with those reported [4,5].

Icariside I (2)

The yellow powder **13** (90 mg) was dissolved in 7.0 M methanolic ammonia (3 mL) and stirred at room temperature for 3 h. The solution was neutralized cautiously with 0.5 M HCl (aq) and left at room temperature for 12 h. The precipitates were collected by filtration and recrystallized from MeOH to furnish **2** as yellow needles. Yield: 58 mg (63%, for 2 steps); mp: 253–255 ºC; $[\alpha]_D^{20}$ = −6.0 (*c* 0.1, CH_3OH). IR (cm^{-1}): 3393, 2918, 1651, 1597, 1557, 1512, 1312, 1260, 1183, 1098. ^1H NMR (400 MHz, DMSO-d_6): δ 12.43 (s, 1H, OH-5), 9.63 (s, 1H, OH-3), 8.14 (d, *J* = 9.0 Hz, 2H, H-2′/6′), 7.14 (d, *J* = 9.0 Hz, 2H, H-3′/5′), 6.60 (s, 1H, H-6), 5.36 (d, *J* = 4.6 Hz, 1H, OH-2‴), 5.20 (t, *J* = 6.9 Hz, 1H, H-2″), 5.14 (d, *J* = 3.9 Hz, 1H, OH-3‴), 5.08 (d, *J* = 5.3 Hz, 1H, OH-4‴), 5.00 (d, *J* = 7.1 Hz, 1H, H-1‴), 4.65 (t, *J* = 5.6 Hz, 1H, OH-6‴), 3.85 (s, 3H, OCH_3-4′), 3.73 – 3.62 (m, 2H, H-1″b/6‴b), 3.50 – 3.44 (m, 3H, H-1″a/5‴/6‴a, overlapped with H_2O), 3.31 – 3.27 (m, 2H, H-2‴/3‴), 3.20 – 3.16 (m, 1H, H-4‴), 1.76 (s, 3H, H-4‴), 1.62 (s, 3H, H-5‴). ^{13}C NMR (100 MHz, DMSO-d_6): δ 176.58 (C-4), 160.71 (C-4′), 160.19 (C-7), 158.63 (C-5), 152.79 (C-9), 146.99 (C-2), 136.27 (C-3), 131.25 (C-3″), 129.44 (C-2′/6′), 123.47 (C-1′), 122.38 (C-2″), 114.21 (C-3′/5′), 108.11 (C-8), 104.55 (C-10), 100.50 (C-1‴), 97.50 (C-6), 77.22 (C-5‴), 76.66 (C-3‴), 73.43 (C-2‴), 69.71 (C-4‴), 60.70 (C-6‴), 55.48 (OCH_3-4′), 25.56 (C-5″), 21.50 (C-1″), 17.99 (C-4″). ESI-HRMS *m*/*z*: 531.1852 $[M+H]^+$ (calcd for $C_{27}H_{31}O_{11}$: 531.1866). The ^1H and ^{13}C NMR data of icariside I (**2**) were in agreement with those reported [6].

References

1. Mei, Q. G.; Wang, C.; Yuan, W. C.; Zhang, G. L. *Beilstein J. Org. Chem.* **2015**, *11*, 288–293.
2. Li, Y.-W.; Li, Y.-X.; Zhang, W.; Guan, H.-S. *Chin*. *J. Org*. *Chem*. **2004**, *24*, 438–439.
3. Nguyen, V.-S.; Shi, L.; Li, Y.; Wang, Q.-A. *Lett. Org. Chem.* **2014**, *11*, 677–681.
4. Dell'Agli, M.; Galli, G. V.; Dal Cero, E.; Belluti, F.; Matera, R.; Zironi, E.; Pagliuca, G.; Bosisio, E. *J. Nat. Prod.* **2008,** *71*, 1513–1517.
5. Liu, R.; Li, A.; Sun, A.; Cui, J.; Kong, L. *J. Chromatogr. A* **2005**, *1064*, 53–57.
6. Han, B.; Shen, T.; Liu, D.; Yang, J. *Chin. Pharm. J.* **2002**, *37*, 740–742.

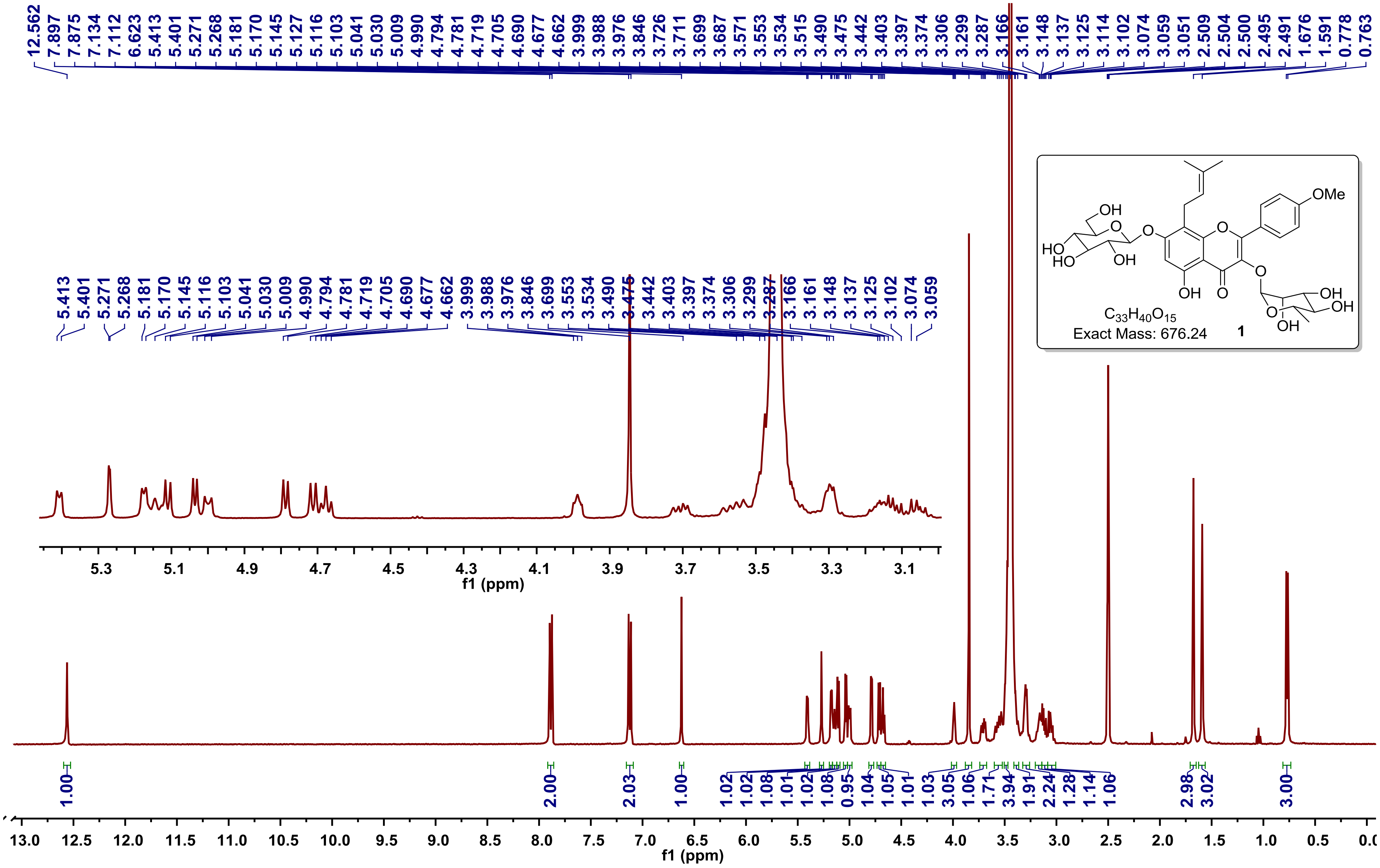
$C_{33}H_{40}O_{15}$
Exact Mass: 676.24
1
f1 (ppm)

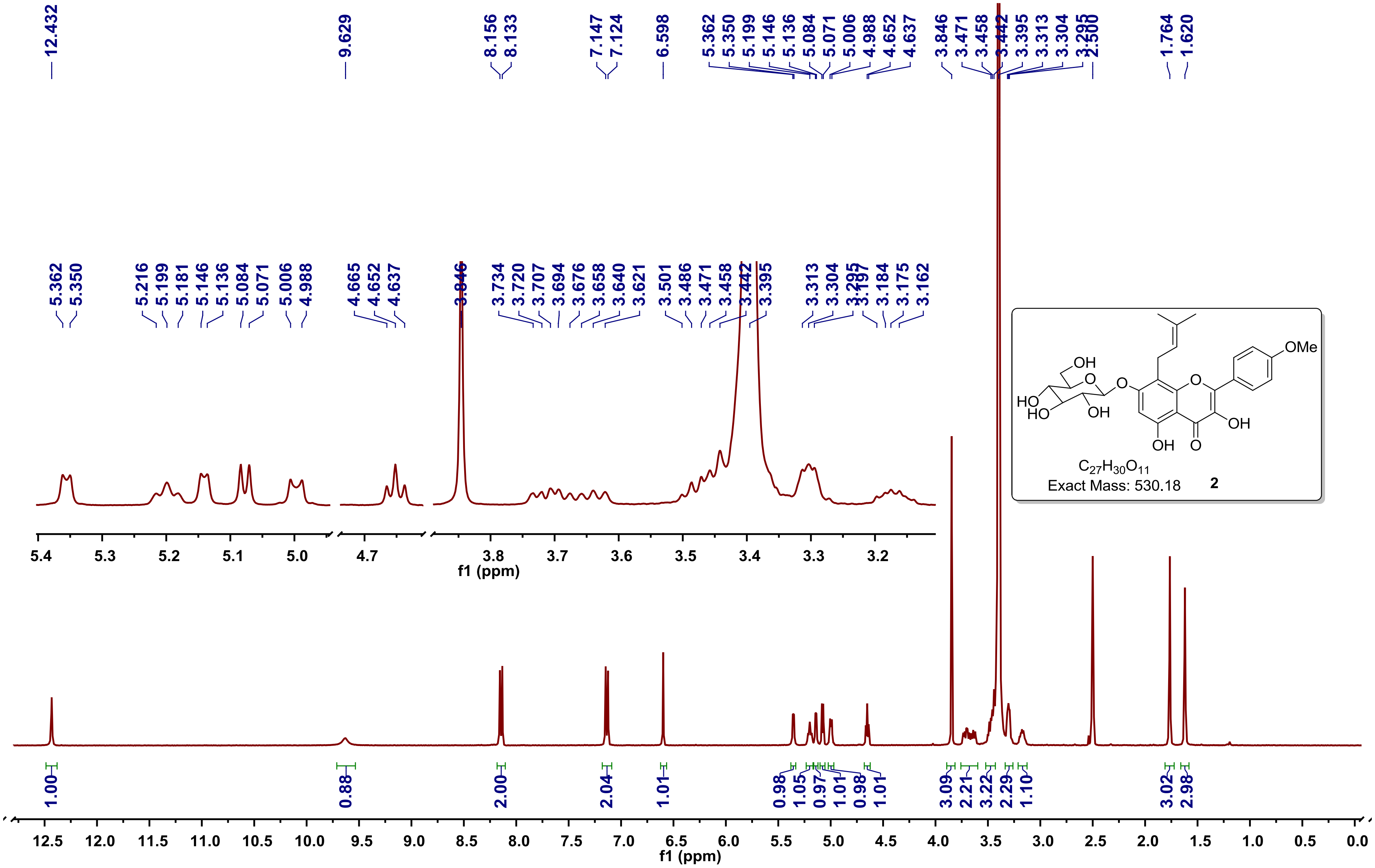
12.432
9.629
8.156
8.133
7.147
7.124
6.598
5.362
5.350
5.199
5.146
5.136
5.084
5.071
5.006
4.988
4.652
4.637
3.846
3.471
3.458
3.442
3.395
3.313
3.304
3.295
2.500
1.764
1.620
5.362
5.350
5.216
5.199
5.181
5.146
5.136
5.084
5.071
5.006
4.988
4.665
4.652
4.637
3.846
3.734
3.720
3.707
3.694
3.676
3.658
3.640
3.621
3.501
3.486
3.471
3.458
3.442
3.395
3.313
3.304
3.295
3.197
3.184
3.175
3.162
5.4 5.3 5.2 5.1 5.0 4.7 3.8 3.7 3.6 3.5 3.4 3.3 3.2
f1 (ppm)
OMe
OH
O
O
O
HO
HO
OH
OH
OH
OH O
$C_{27}H_{30}O_{11}$
Exact Mass: 530.18
2
1.00
0.88
2.00
2.04
1.01
0.98
1.05
0.97
1.01
0.98
1.01
3.09
2.21
3.22
2.29
1.10
3.02
2.98
12.5 12.0 11.5 11.0 10.5 10.0 9.5 9.0 8.5 8.0 7.5 7.0 6.5 6.0 5.5 5.0 4.5 4.0 3.5 3.0 2.5 2.0 1.5 1.0 0.5 0.0
f1 (ppm)

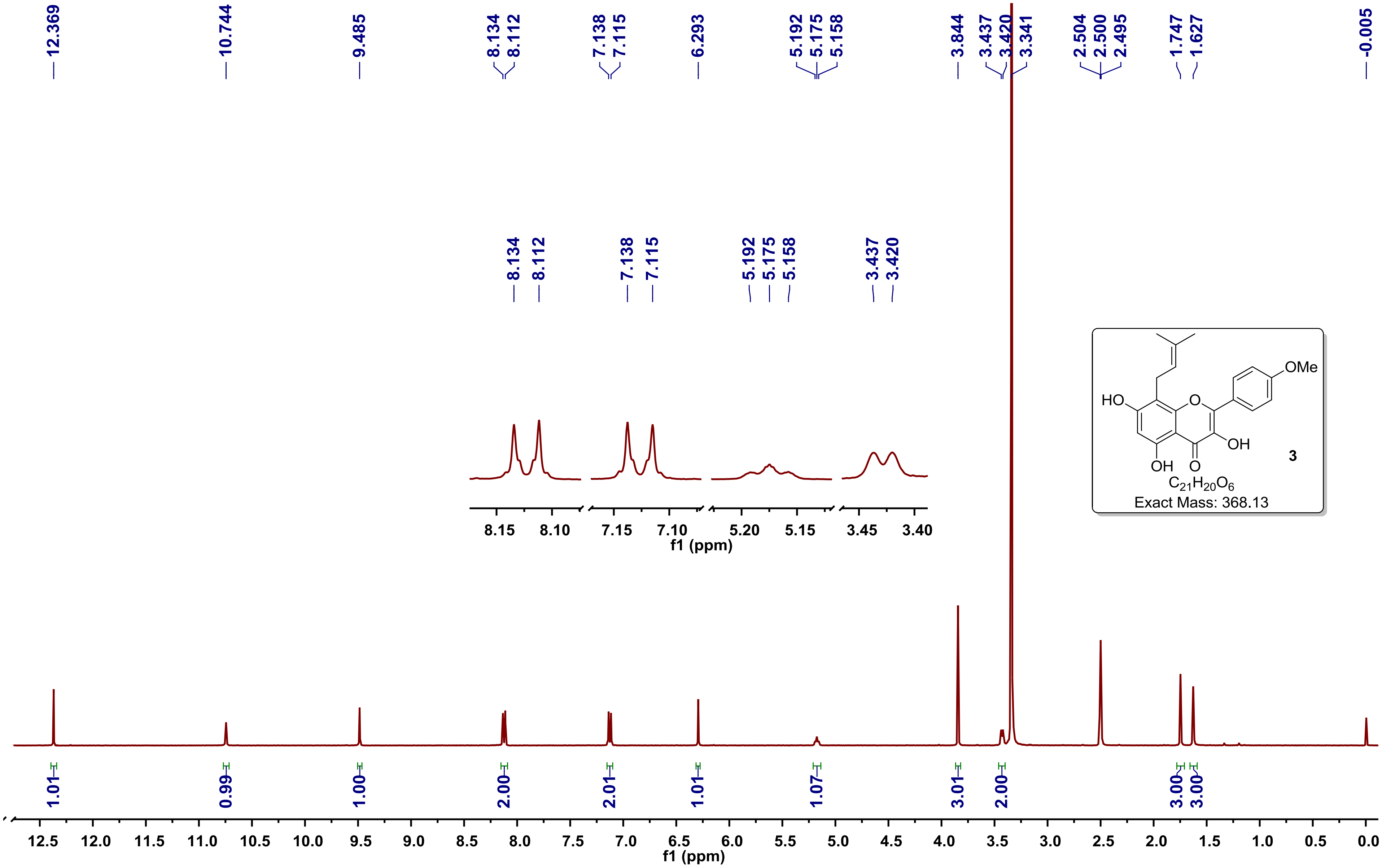
12.369
10.744
9.485
8.134
8.112
7.138
7.115
6.293
5.192
5.175
5.158
3.844
3.437
3.420
3.341
2.504
2.500
2.495
1.747
1.627
-0.005
1.01
0.99
1.00
2.00
2.01
1.01
1.07
3.01
2.00
3.00
3.00
f1 (ppm)
HO
O
OMe
OH
OH
O
3
$C_{21}H_{20}O_6$
Exact Mass: 368.13

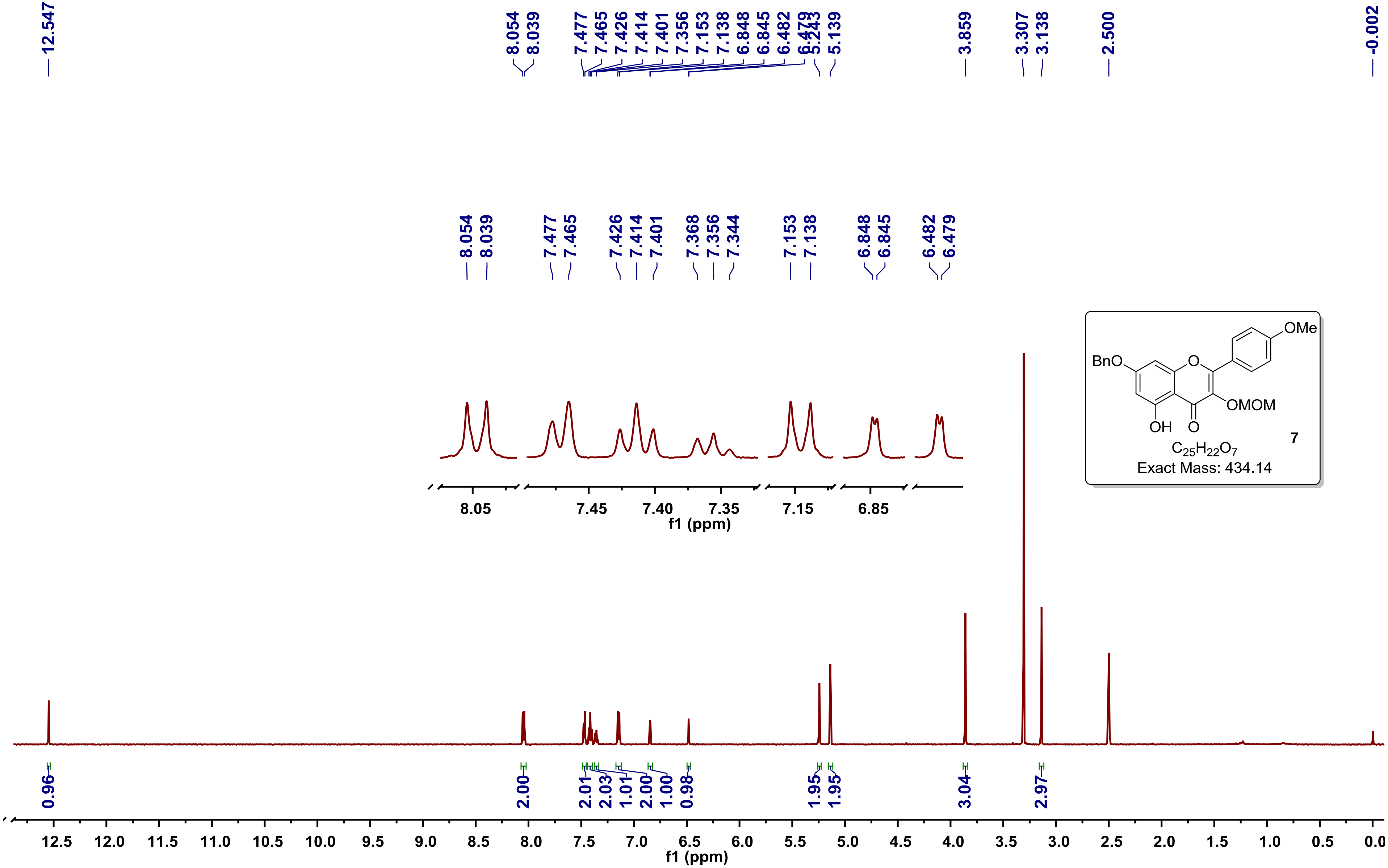
12.547
8.054
8.039
7.477
7.465
7.426
7.414
7.401
7.368
7.356
7.344
7.153
7.138
6.848
6.845
6.482
6.479
5.243
5.139
3.859
3.307
3.138
2.500
-0.002
BnO
O
OMe
OMOM
OH
O
7
$C_{25}H_{22}O_7$
Exact Mass: 434.14
0.96
2.00
2.01
2.03
1.01
2.00
1.00
0.98
1.95
1.95
3.04
2.97
f1 (ppm)

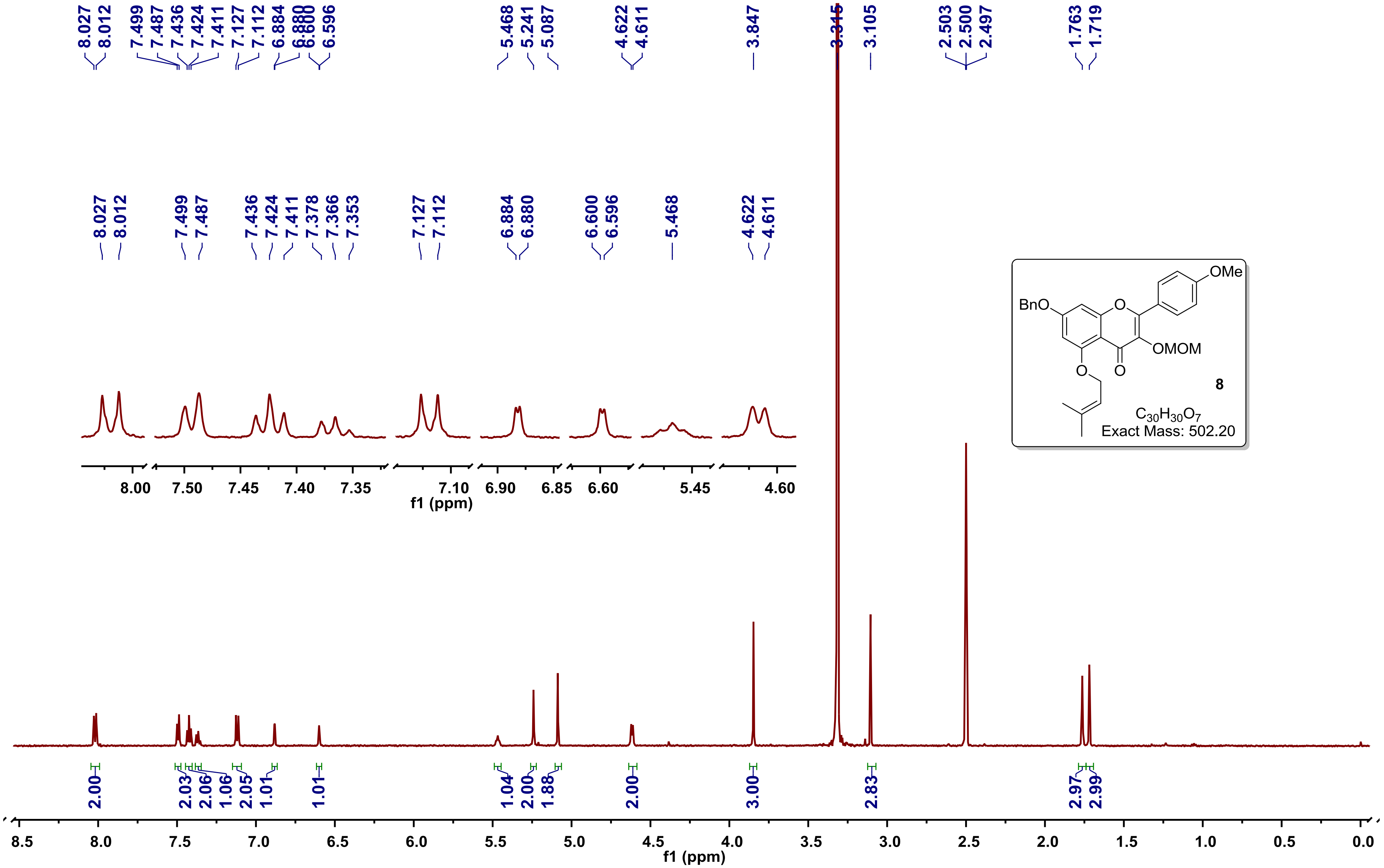
8.027
8.012
7.499
7.487
7.436
7.424
7.411
7.127
7.112
6.884
6.880
6.600
6.596
5.468
5.241
5.087
4.622
4.611
3.847
3.315
3.105
2.503
2.500
2.497
1.763
1.719
7.378
7.366
7.353
BnO
O
OMe
OMOM
O
O
8
$C_{30}H_{30}O_7$
Exact Mass: 502.20
8.00
7.50
7.45
7.40
7.35
7.10
6.90
6.85
6.60
5.45
4.60
f1 (ppm)
2.00
2.03
2.06
1.06
2.05
1.01
1.01
1.04
2.00
1.88
2.00
3.00
2.83
2.97
2.99
8.5
8.0
7.5
7.0
6.5
6.0
5.5
5.0
4.5
4.0
3.5
3.0
2.5
2.0
1.5
1.0
0.5
0.0
f1 (ppm)

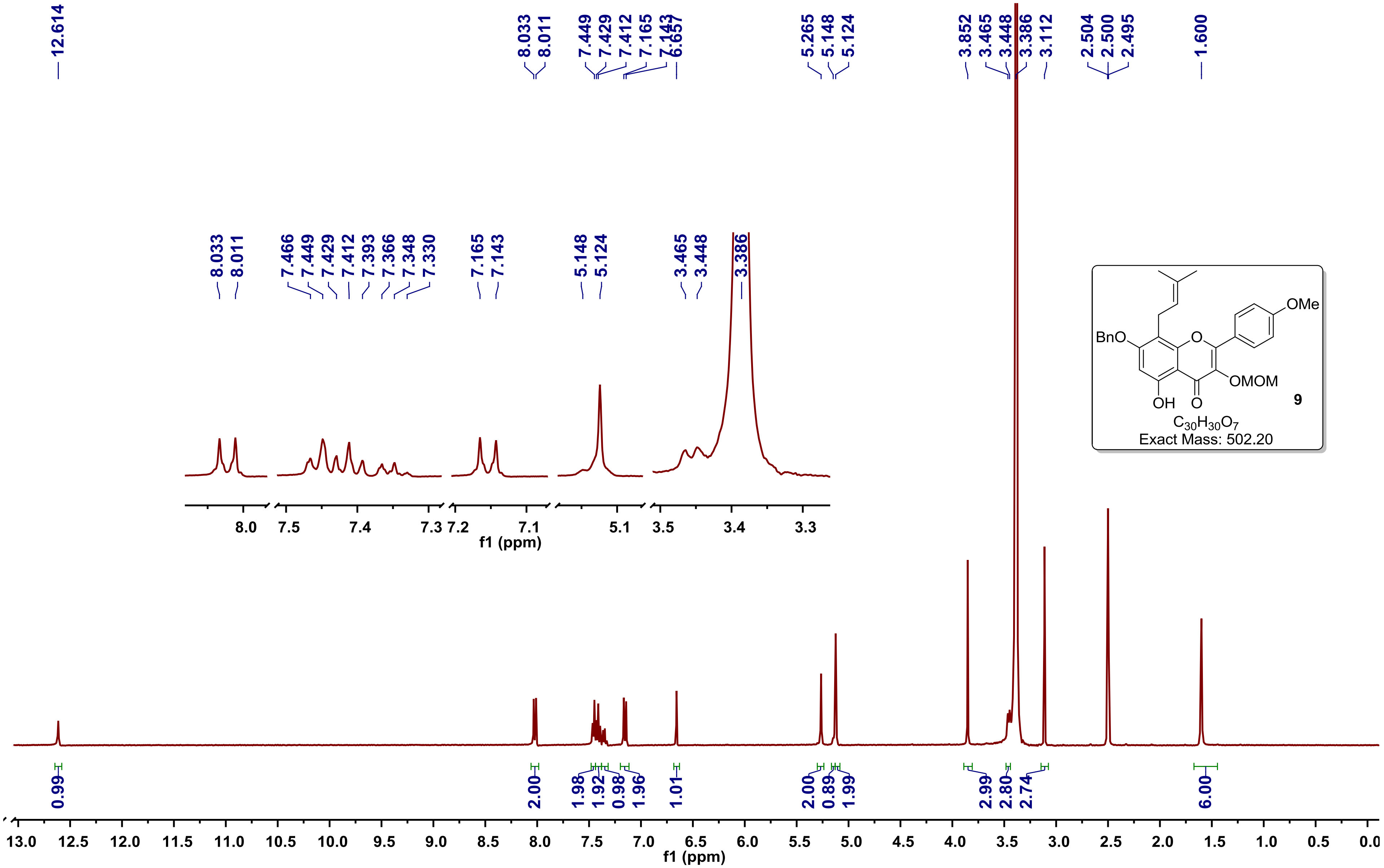
12.614
8.033
8.011
7.449
7.429
7.412
7.165
7.143
6.657
5.265
5.148
5.124
3.852
3.465
3.448
3.386
3.112
2.504
2.500
2.495
1.600
7.466
7.393
7.366
7.348
7.330
BnO
O
OMe
OMOM
OH
9
$C_{30}H_{30}O_7$
Exact Mass: 502.20
f1 (ppm)
0.99
2.00
1.98
1.92
0.98
1.96
1.01
2.00
0.89
1.99
2.99
2.80
2.74
6.00

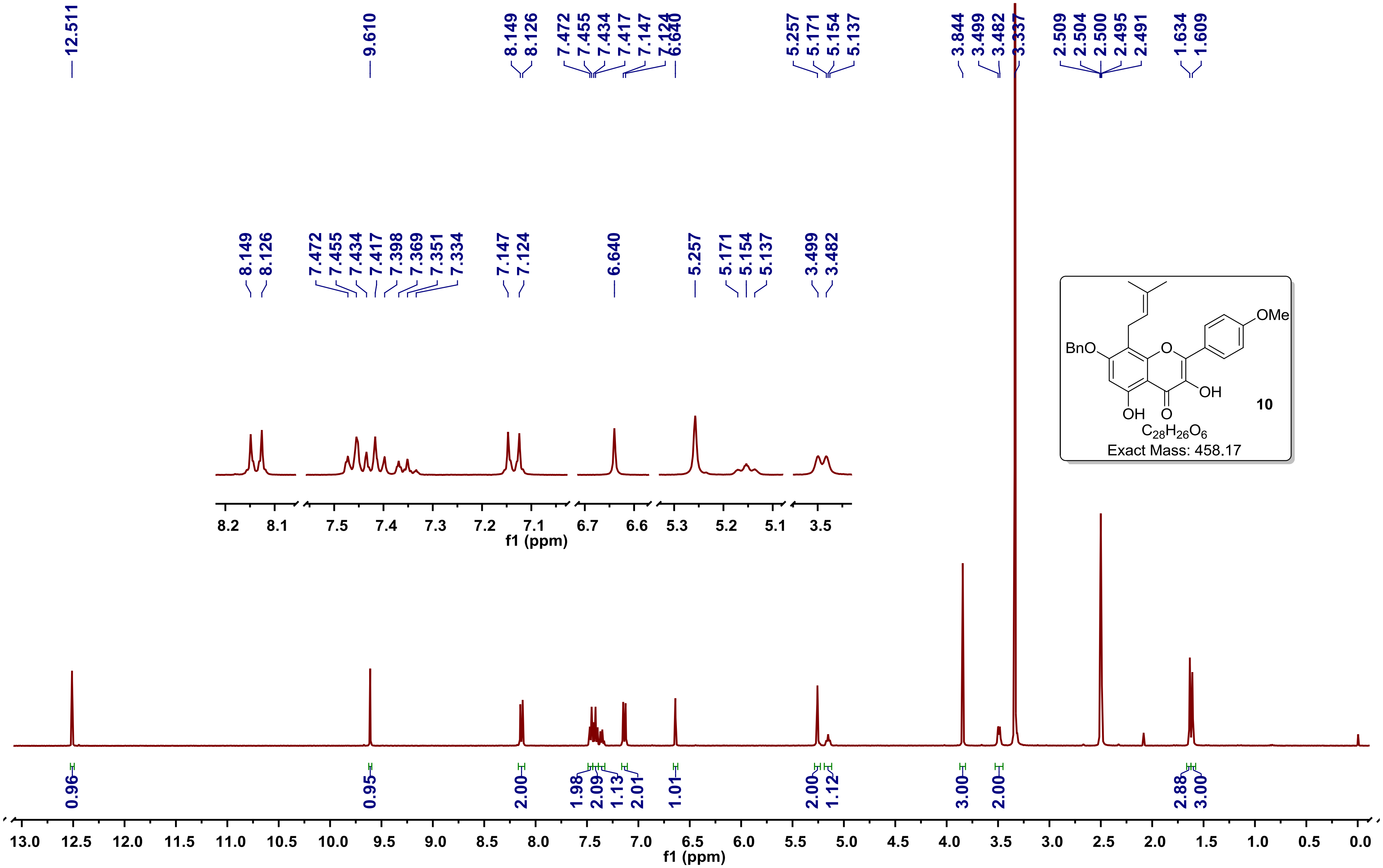

12.511
9.610
8.149
8.126
7.472
7.455
7.434
7.417
7.147
7.124
6.640
5.257
5.171
5.154
5.137
3.844
3.499
3.482
3.337
2.509
2.504
2.500
2.495
2.491
1.634
1.609
BnO
OMe
O
OH
OH
O
10
$C_{28}H_{26}O_6$
Exact Mass: 458.17
f1 (ppm)
0.96
0.95
2.00
1.98
2.09
1.13
2.01
1.01
2.00
1.12
3.00
2.00
2.88
3.00

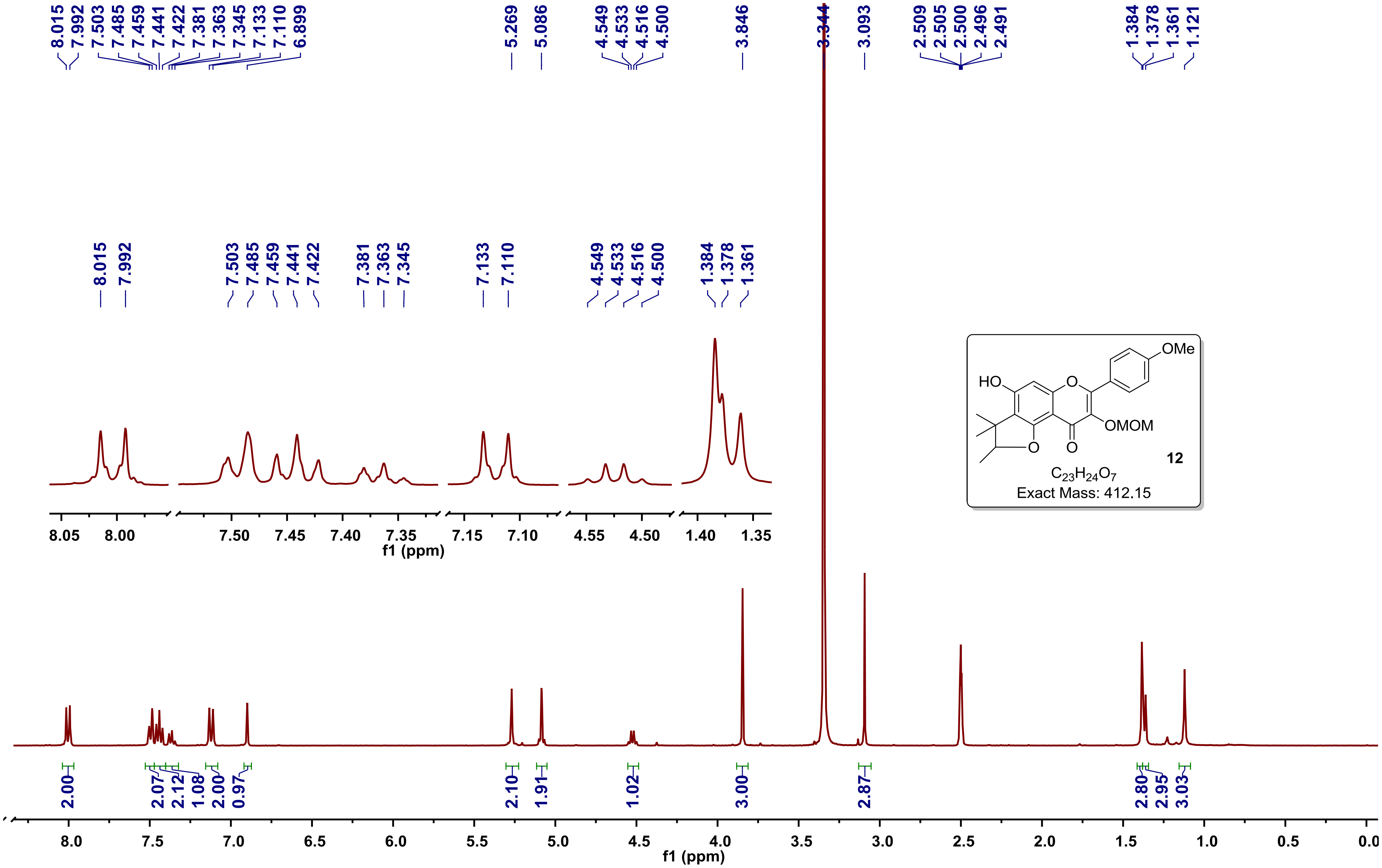
8.015
7.992
7.503
7.485
7.459
7.441
7.422
7.381
7.363
7.345
7.133
7.110
6.899
5.269
5.086
4.549
4.533
4.516
4.500
3.846
3.344
3.093
2.509
2.505
2.500
2.496
2.491
1.384
1.378
1.361
1.121
HO
OMe
O
OMOM
O
O
12
$C_{23}H_{24}O_7$
Exact Mass: 412.15
f1 (ppm)
2.00
2.07
2.12
1.08
2.00
0.97
2.10
1.91
1.02
3.00
2.87
2.80
2.95
3.03
f1 (ppm)

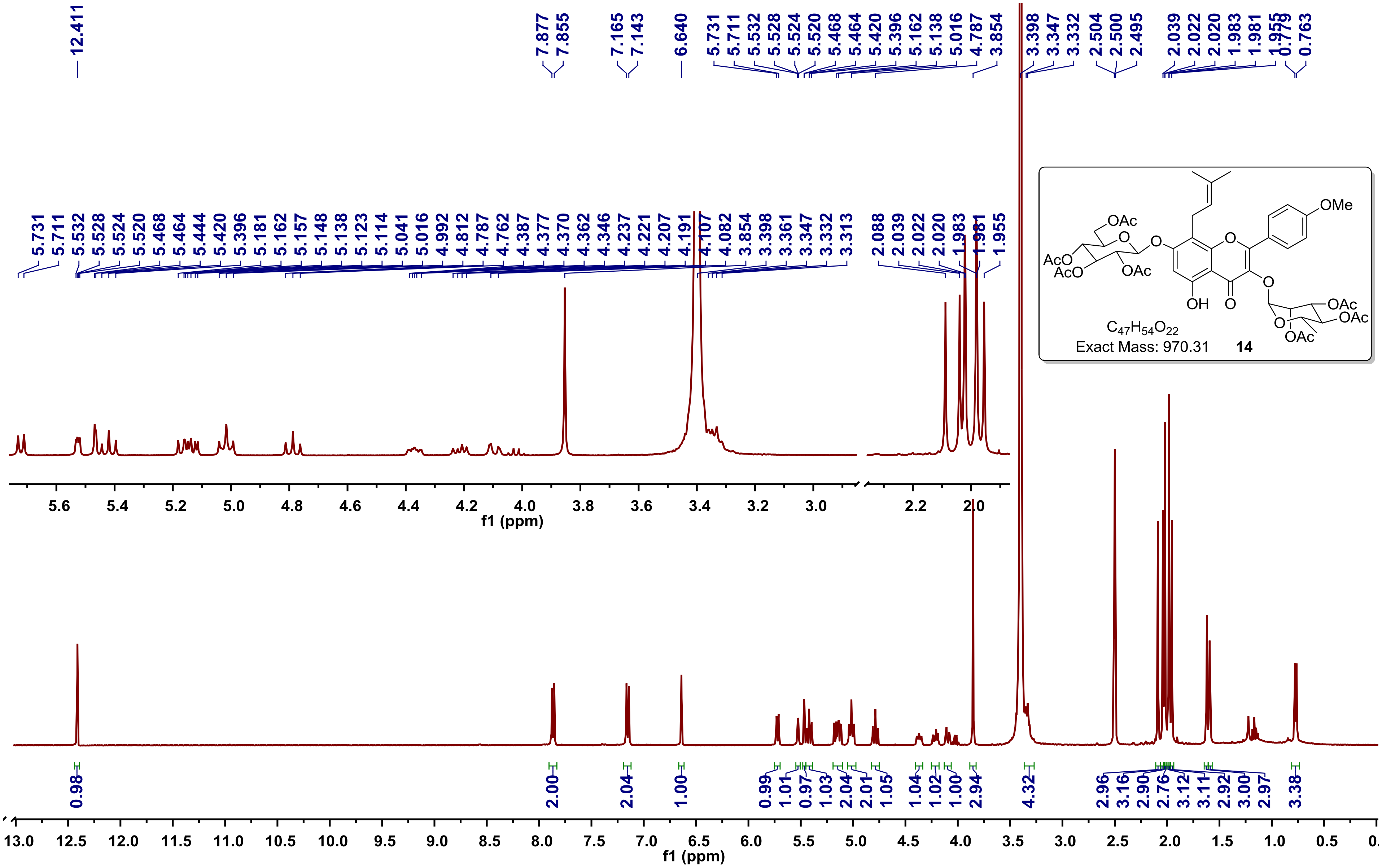

C47H54O22
Exact Mass: 970.31
14
f1 (ppm)
f1 (ppm)

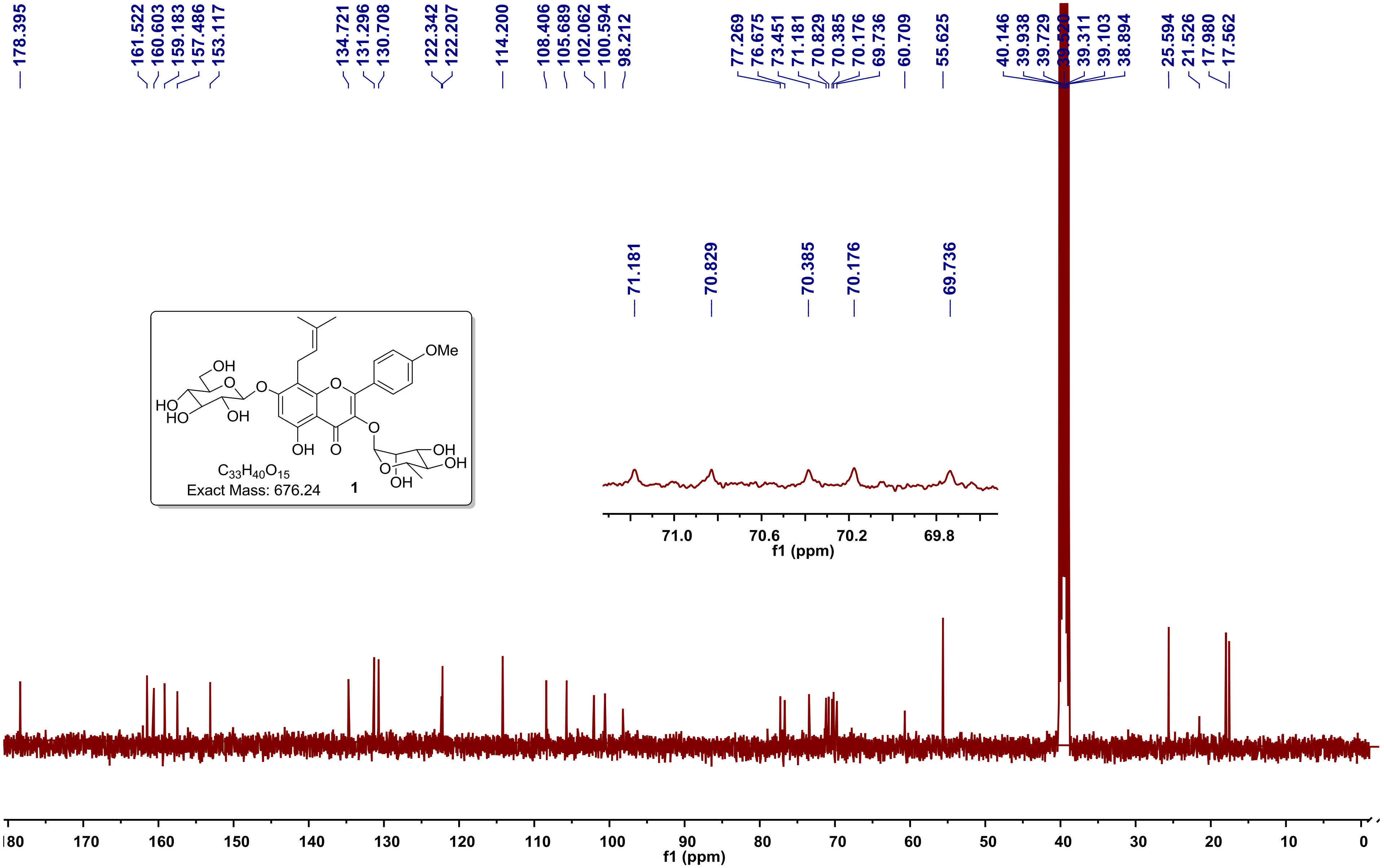

178.395
161.522
160.603
159.183
157.486
153.117
134.721
131.296
130.708
122.342
122.207
114.200
108.406
105.689
102.062
100.594
98.212
77.269
76.675
73.451
71.181
70.829
70.385
70.176
69.736
60.709
55.625
40.146
39.938
39.729
39.520
39.311
39.103
38.894
25.594
21.526
17.980
17.562
71.181
70.829
70.385
70.176
69.736
71.0
70.6
70.2
69.8
f1 (ppm)
OMe
OH
O
HO
HO
OH
OH
C33H40O15
Exact Mass: 676.24
1
180
170
160
150
140
130
120
110
100
90
80
70
60
50
40
30
20
10
0
f1 (ppm)

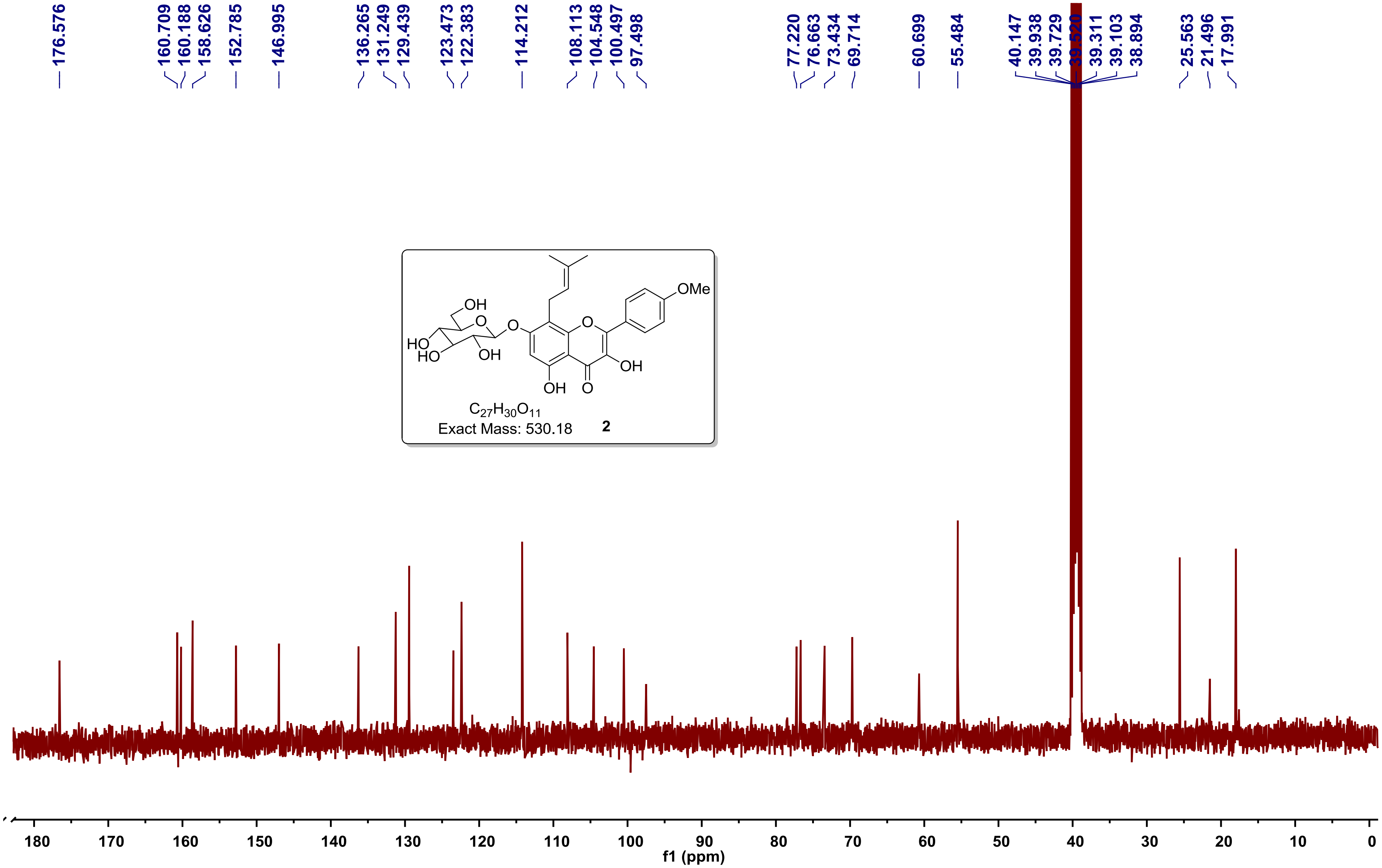
176.576
160.709
160.188
158.626
152.785
146.995
136.265
131.249
129.439
123.473
122.383
114.212
108.113
104.548
100.497
97.498
77.220
76.663
73.434
69.714
60.699
55.484
40.147
39.938
39.729
39.520
39.311
39.103
38.894
25.563
21.496
17.991
OMe
OH
O
HO
HO
OH
OH
OH
O
C27H30O11
Exact Mass: 530.18
2
f1 (ppm)

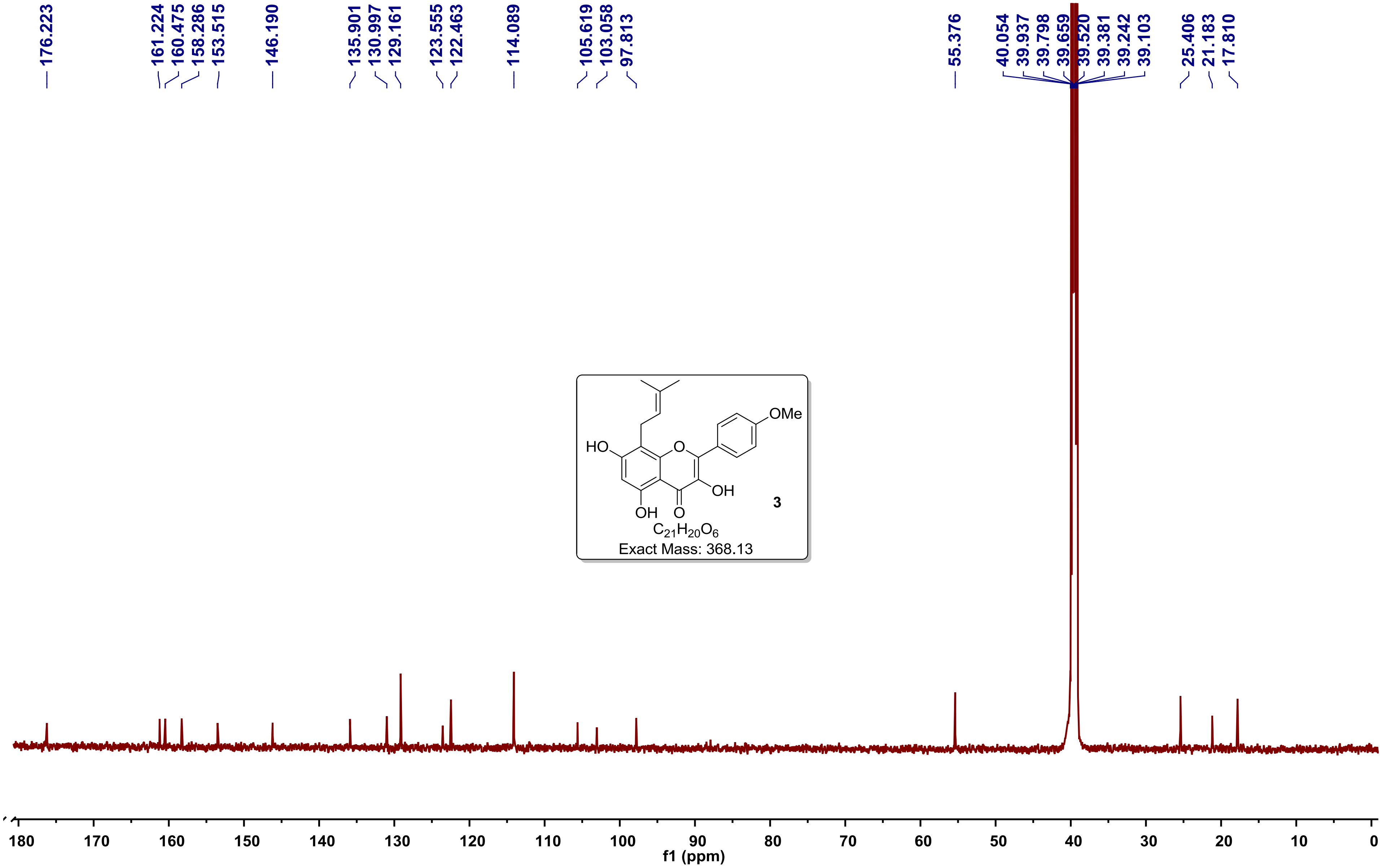
176.223
161.224
160.475
158.286
153.515
146.190
135.901
130.997
129.161
123.555
122.463
114.089
105.619
103.058
97.813
55.376
40.054
39.937
39.798
39.659
39.520
39.381
39.242
39.103
25.406
21.183
17.810
HO
O
OMe
OH
OH
O
3
$C_{21}H_{20}O_6$
Exact Mass: 368.13
180
170
160
150
140
130
120
110
100
90
80
70
60
50
40
30
20
10
0
f1 (ppm)

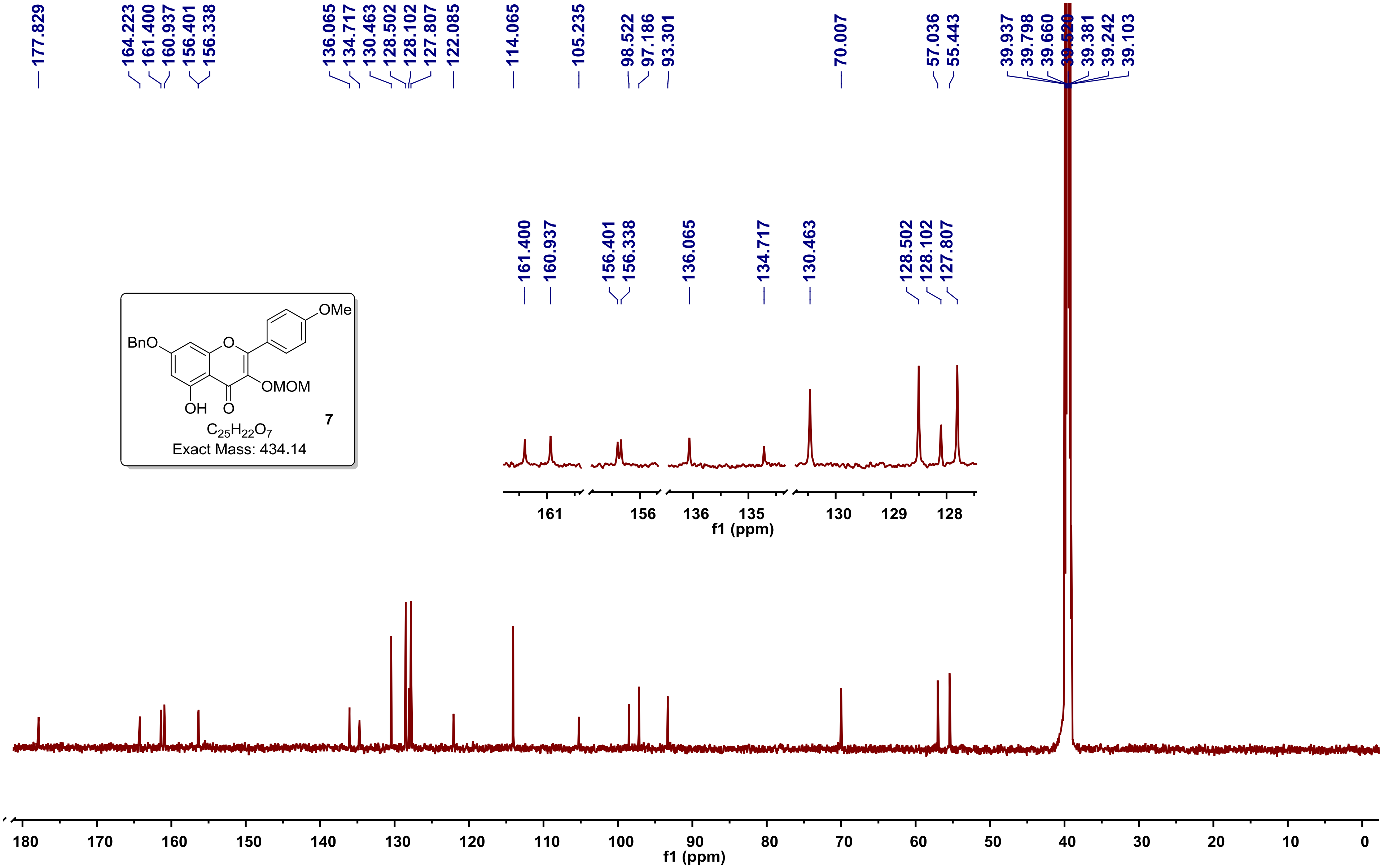
177.829
164.223
161.400
160.937
156.401
156.338
136.065
134.717
130.463
128.502
128.102
127.807
122.085
114.065
105.235
98.522
97.186
93.301
70.007
57.036
55.443
39.937
39.798
39.660
39.520
39.381
39.242
39.103
OMe
BnO
O
OMOM
OH
O
7
C25H22O7
Exact Mass: 434.14
f1 (ppm)
f1 (ppm)

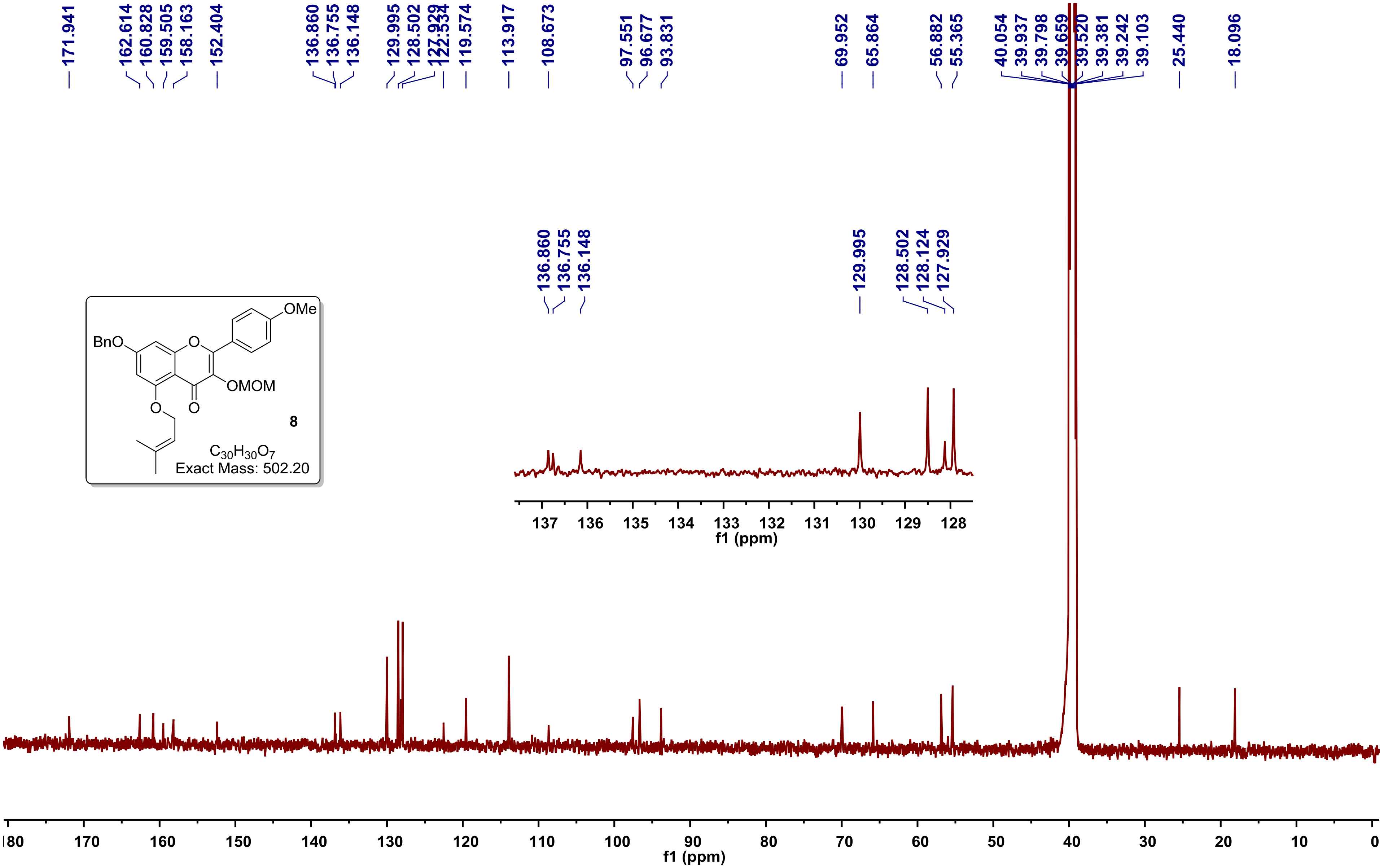

8

$C_{30}H_{30}O_7$
Exact Mass: 502.20

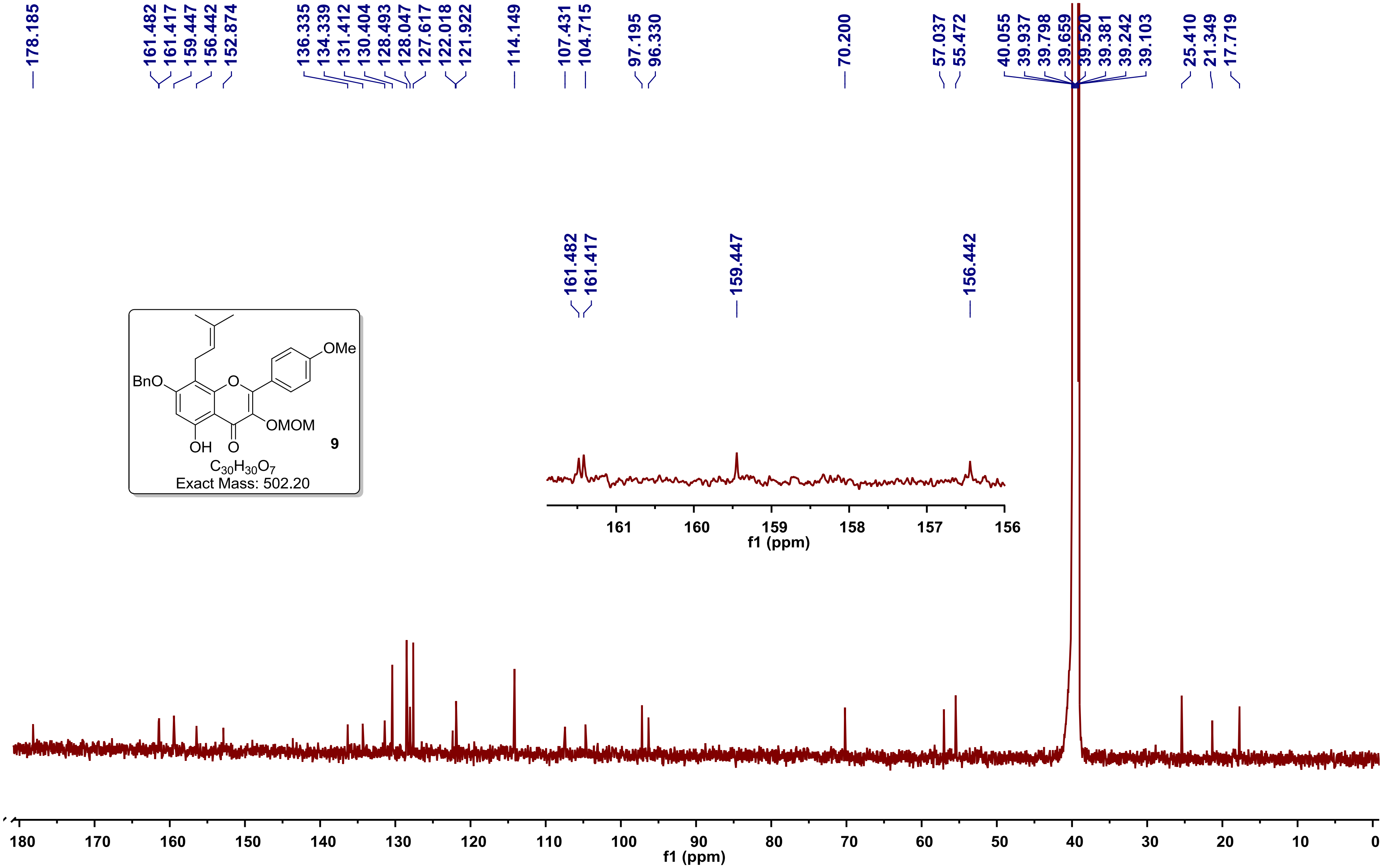
178.185
161.482
161.417
159.447
156.442
152.874
136.335
134.339
131.412
130.404
128.493
128.047
127.617
122.018
121.922
114.149
107.431
104.715
97.195
96.330
70.200
57.037
55.472
40.055
39.937
39.798
39.659
39.520
39.381
39.242
39.103
25.410
21.349
17.719
BnO
OMe
O
OMOM
OH
O
9
C30H30O7
Exact Mass: 502.20
f1 (ppm)
f1 (ppm)

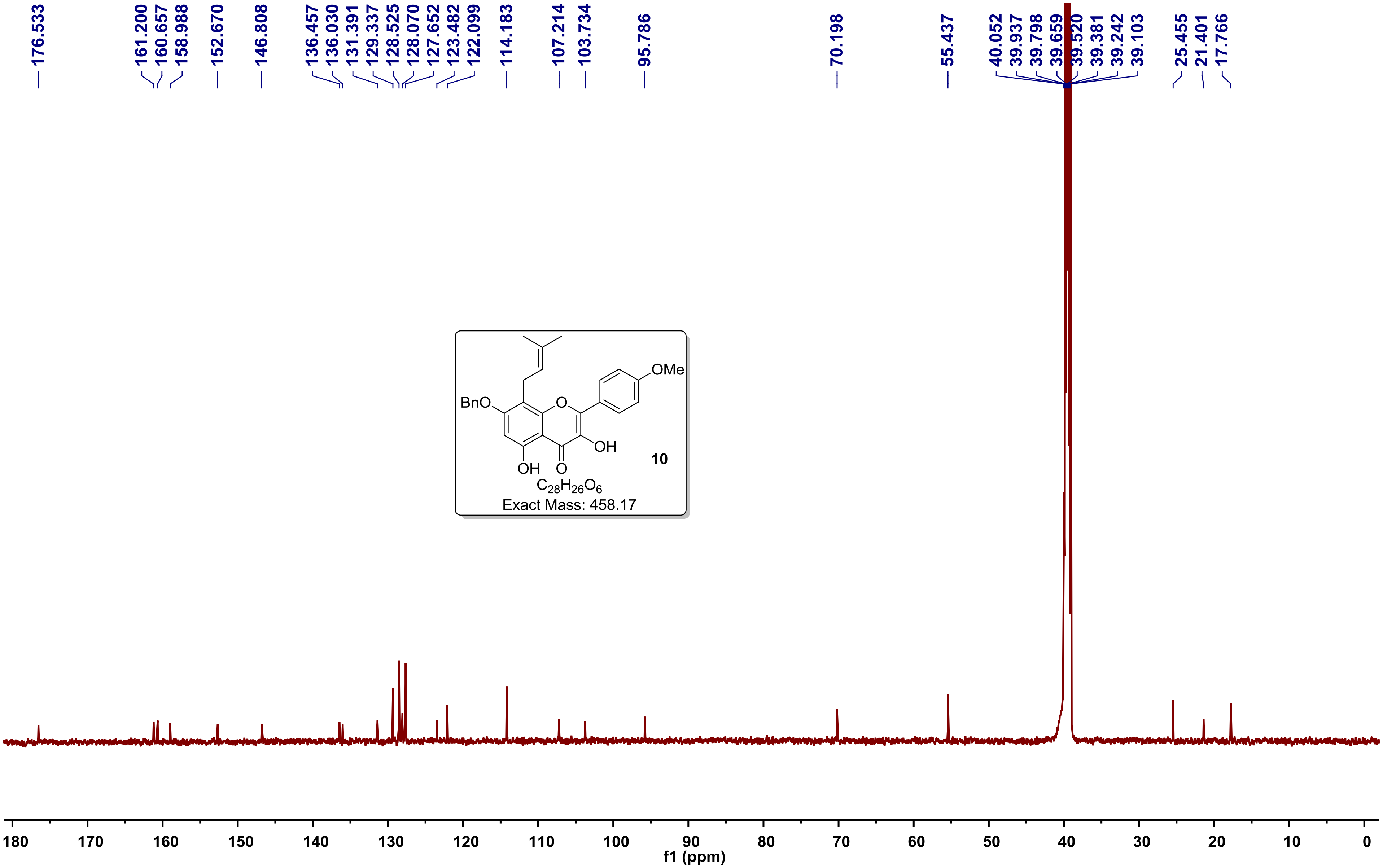
176.533
161.200
160.657
158.988
152.670
146.808
136.457
136.030
131.391
129.337
128.525
128.070
127.652
123.482
122.099
114.183
107.214
103.734
95.786
70.198
55.437
40.052
39.937
39.798
39.659
39.520
39.381
39.242
39.103
25.455
21.401
17.766
OMe
BnO
O
OH
OH
O
10
$C_{28}H_{26}O_6$
Exact Mass: 458.17
180
170
160
150
140
130
120
110
100
90
80
70
60
50
40
30
20
10
0
f1 (ppm)

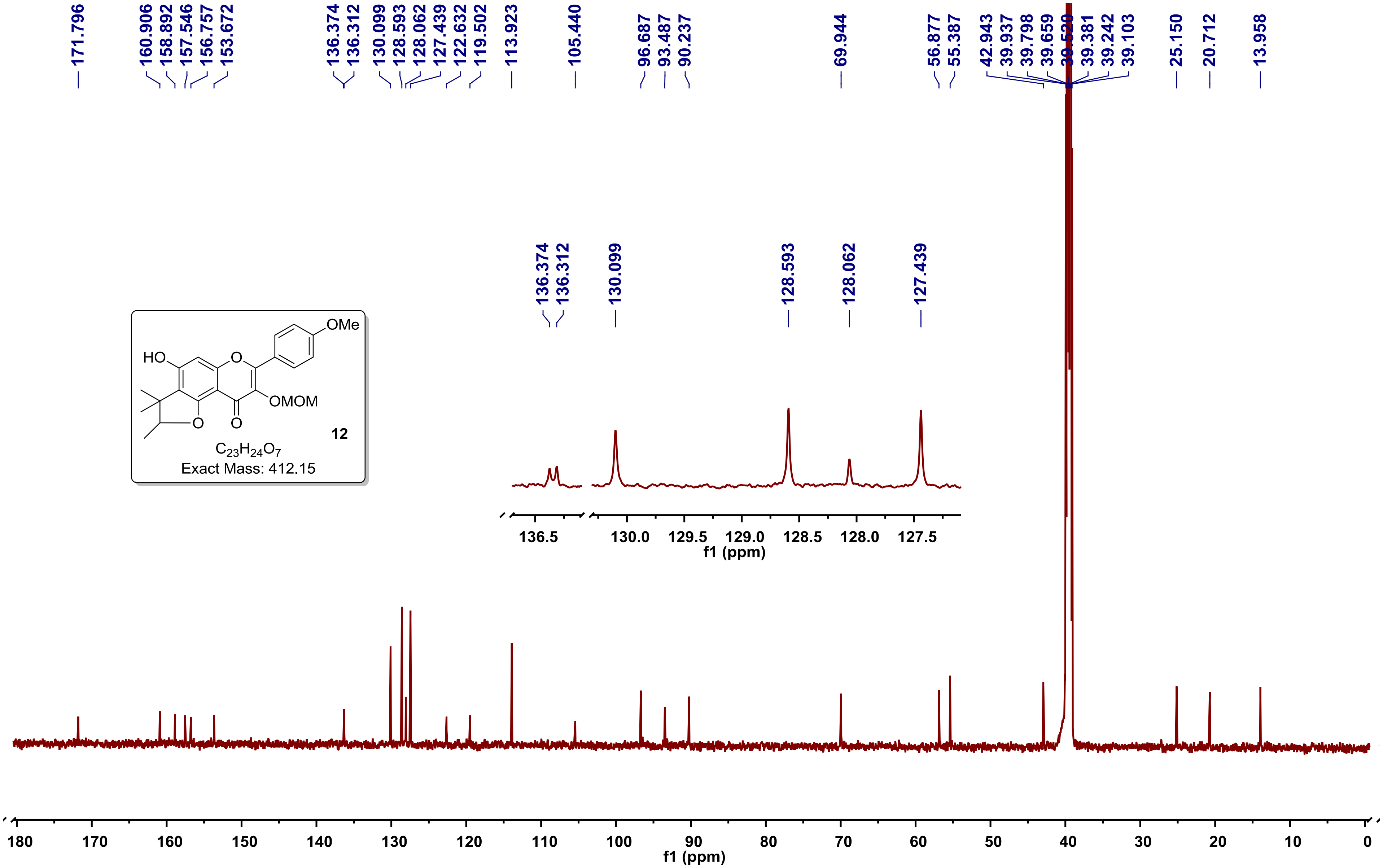
171.796
160.906
158.892
157.546
156.757
153.672
136.374
136.312
130.099
128.593
128.062
127.439
122.632
119.502
113.923
105.440
96.687
93.487
90.237
69.944
56.877
55.387
42.943
39.937
39.798
39.659
39.520
39.381
39.242
39.103
25.150
20.712
13.958
HO
O
OMe
OMOM
O
O
12
$C_{23}H_{24}O_7$
Exact Mass: 412.15
136.5
130.0
129.5
129.0
128.5
128.0
127.5
f1 (ppm)
180
170
160
150
140
130
120
110
100
90
80
70
60
50
40
30
20
10
0
f1 (ppm)

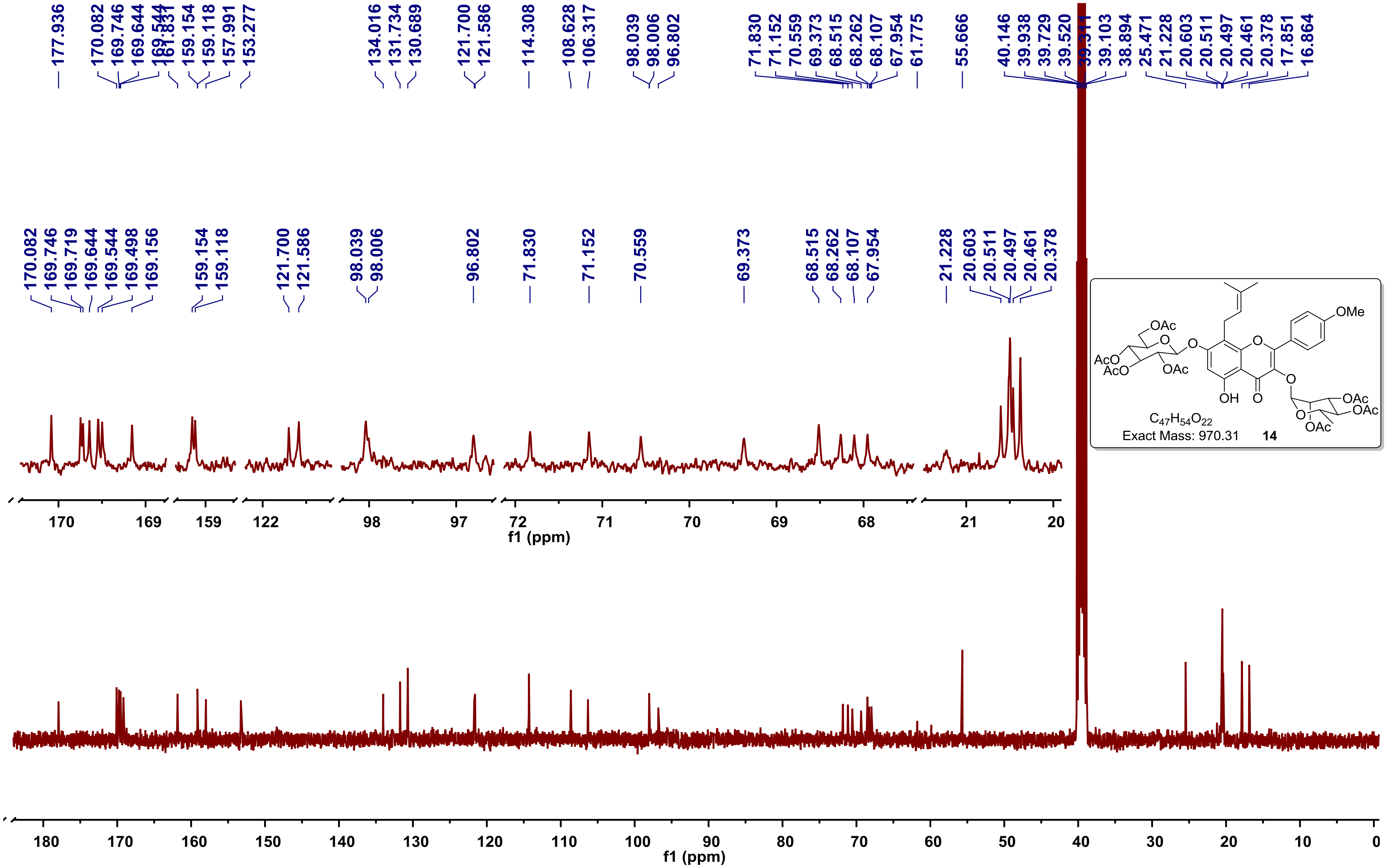

177.936 170.082 169.746 169.644 169.544 161.831 159.154 159.118 157.991 153.277 134.016 131.734 130.689 121.700 121.586 114.308 108.628 106.317 98.039 98.006 96.802 71.830 71.152 70.559 69.373 68.515 68.262 68.107 67.954 61.775 55.666 40.146 39.938 39.729 39.520 39.311 39.103 38.894 25.471 21.228 20.603 20.511 20.497 20.461 20.378 17.851 16.864
170.082 169.746 169.719 169.644 169.544 169.498 169.156 159.154 159.118 121.700 121.586 98.039 98.006 96.802 71.830 71.152 70.559 69.373 68.515 68.262 68.107 67.954 21.228 20.603 20.511 20.497 20.461 20.378
OMe
OAc
AcO
AcO
OAc
OH
OAc
OAc
OAc
C47H54O22
Exact Mass: 970.31
14
f1 (ppm)
f1 (ppm)

Mass Spectrum SmartFormula Report

Analysis Info

Analysis Name	D:\Data\USER-2015\m4.d	Acquisition Date	1/21/2015 3:40:00 PM
Method	lc-ms-ljr-20111104.m	Operator	Ma
Sample Name	m4	Instrument / Ser#	micrOTOF-Q II 10203
Comment			

Acquisition Parameter

Source Type	ESI	Ion Polarity	Positive	Set Nebulizer	0.4 Bar		
Focus	Not active	Set Capillary	4500 V	Set Dry Heater	180 °C		
Scan Begin	50 m/z	Set End Plate Offset	-500 V	Set Dry Gas	3.0 l/min		
Scan End	1500 m/z	Set Collision Cell RF	150.0 Vpp	Set Divert Valve	Source		

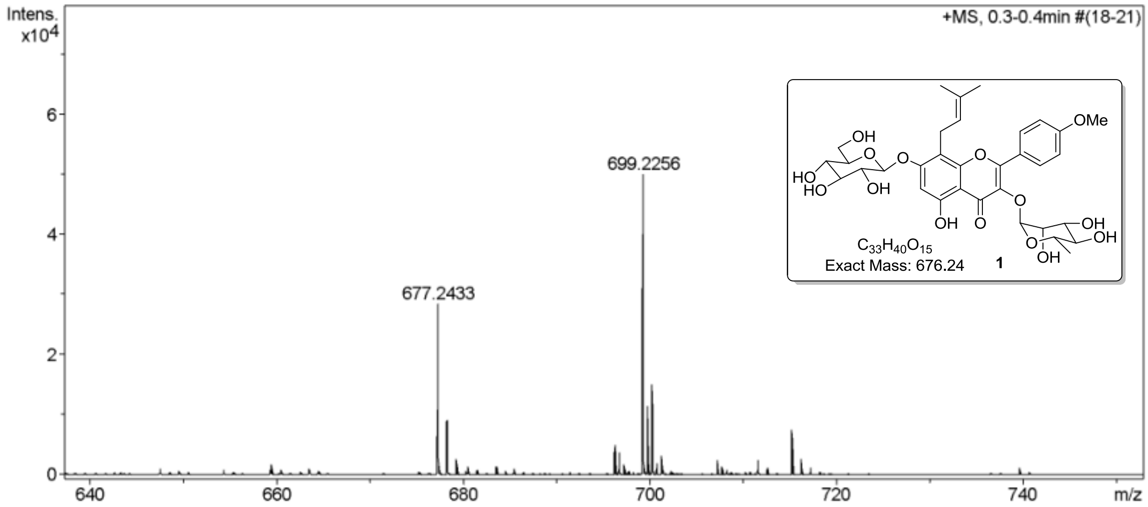

Meas. m/z	#	Formula	Score	m/z	err [ppm]	Mean err [ppm]	mSigma	rdb	e⁻ Conf	N-Rule
677.2433	1	C 33 H 41 O 15	100.00	677.2440	1.0	1.3	25.7	13.5	even	ok
699.2256	1	C 33 H 40 Na O 15	100.00	699.2259	0.5	0.8	38.1	13.5	even	ok

Elemental Composition Report

Single Mass Analysis
Sample Name: mei 530
Tolerance = 5.0 PPM
RDB: min = -10.0, max = 120.0
Selected filters: None

Monoisotopic Mass, Odd and Even Electron Ions
Elements Used:
C: 0-40 H: 0-100 O: 0-20 N: 0-10

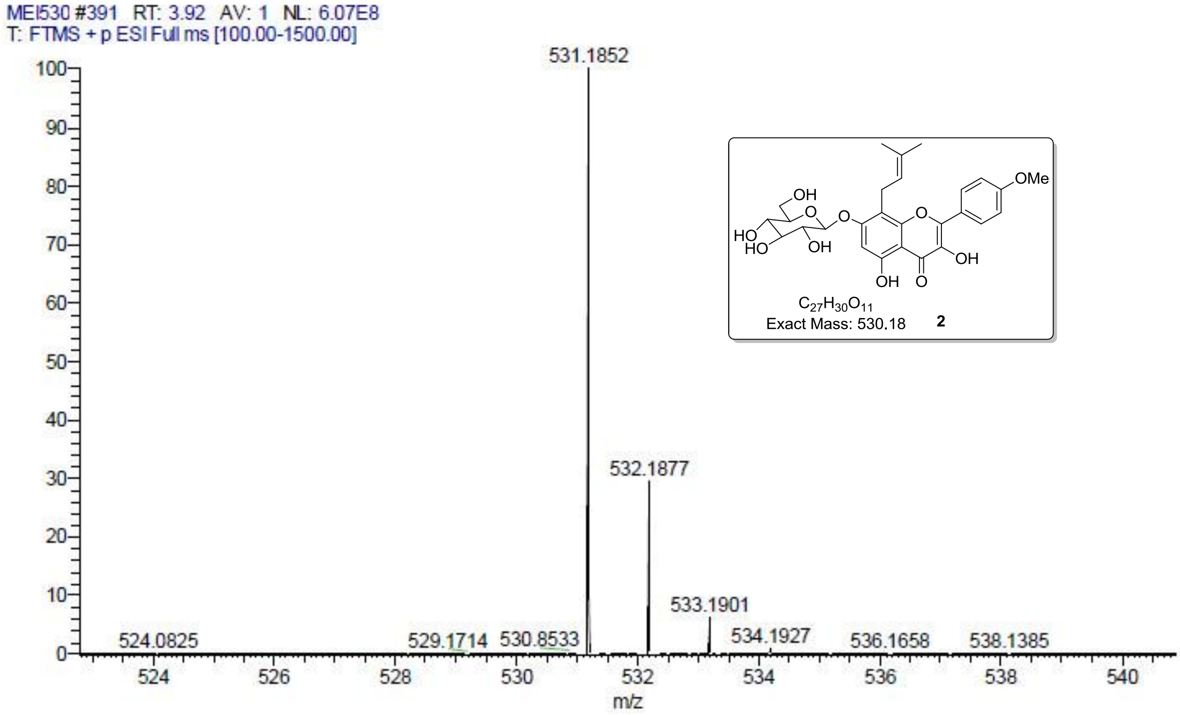

Measured Mass: 531.1852

Idx	Formula	RDB	Delta ppm
1	C27 H30 O11	12.5	-1.170

Mass Spectrum SmartFormula Report

Analysis Info

Analysis Name	D:\Data\USER-2013\mei368.d	Acquisition Date	10/15/2013 11:43:16 AM
Method	WU_tune_low_20121222.m	Operator	Ma
Sample Name	mei368	Instrument / Ser#	micrOTOF-Q II 10203
Comment			

Acquisition Parameter

Source Type	ESI	Ion Polarity	Positive	Set Nebulizer	0.4 Bar
Focus	Not active	Set Capillary	4500 V	Set Dry Heater	100 °C
Scan Begin	50 m/z	Set End Plate Offset	-500 V	Set Dry Gas	3.0 l/min
Scan End	3000 m/z	Set Collision Cell RF	150.0 Vpp	Set Divert Valve	Source

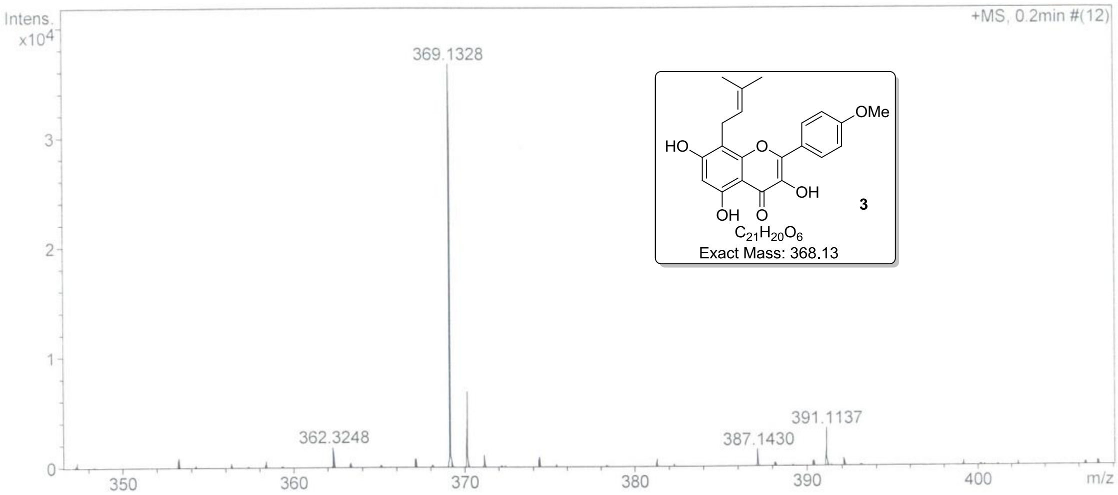

3

$C_{21}H_{20}O_6$
Exact Mass: 368.13

Meas. m/z	#	Formula	Score	m/z	err [ppm]	Mean err [ppm]	mSigma	rdb	e⁻ Conf	N-Rule
369.1328	1	C 21 H 21 O 6	100.00	369.1333	1.4	1.7	31.4	11.5	even	ok

Bruker Compass DataAnalysis 4.0 printed: 10/15/2013 11:55:22 AM Page 1 of 1

Mass Spectrum SmartFormula Report

Analysis Info

Analysis Name	D:\Data\USER-2013\M-434.d	Acquisition Date	5/14/2013 4:04:15 PM
Method	WU_tune_low_20121222.m	Operator	Ma
Sample Name	M-434	Instrument / Ser#	micrOTOF-Q II 10203
Comment			

Acquisition Parameter

Source Type	ESI	Ion Polarity	Positive	Set Nebulizer	0.3 Bar
Focus	Not active	Set Capillary	4500 V	Set Dry Heater	180 °C
Scan Begin	50 m/z	Set End Plate Offset	-500 V	Set Dry Gas	4.0 l/min
Scan End	3000 m/z	Set Collision Cell RF	150.0 Vpp	Set Divert Valve	Source

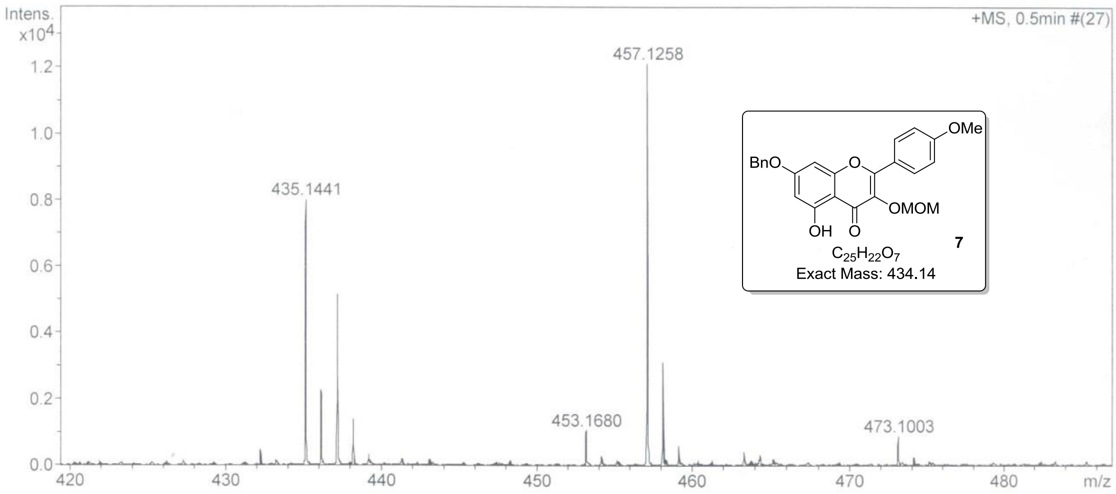

Meas. m/z	#	Formula	Score	m/z	err [ppm]	Mean err [ppm]	mSigma	rdb	e⁻ Conf	N-Rule
457.1258	1	C 25 H 22 Na O 7	100.00	457.1258	-0.1	0.1	7.0	14.5	even	ok

Mass Spectrum SmartFormula Report

Analysis Info

Analysis Name	D:\Data\USER-2013\MEI502W.d	Acquisition Date	5/28/2013 4:33:47 PM
Method	WU_tune_low_20121222.m	Operator	Ma
Sample Name	MEI502W	Instrument / Ser#	micrOTOF-Q II 10203
Comment			

Acquisition Parameter

Source Type	ESI	Ion Polarity	Positive	Set Nebulizer	0.3 Bar
Focus	Not active	Set Capillary	4500 V	Set Dry Heater	180 °C
Scan Begin	50 m/z	Set End Plate Offset	-500 V	Set Dry Gas	4.0 l/min
Scan End	3000 m/z	Set Collision Cell RF	150.0 Vpp	Set Divert Valve	Source

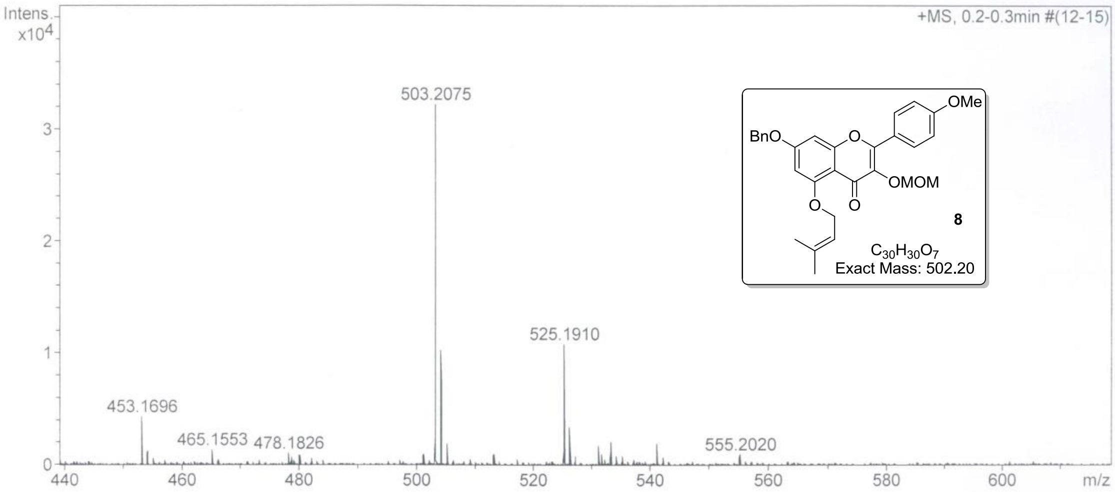

Meas. m/z	#	Formula	Score	m/z	err [ppm]	Mean err [ppm]	mSigma	rdb	e⁻ Conf	N-Rule
503.2075	1	C 30 H 31 O 7	100.00	503.2064	-2.1	-2.9	6.8	15.5	even	ok

Bruker Compass DataAnalysis 4.0 printed: 5/28/2013 4:39:15 PM Page 1 of 1

Mass Spectrum SmartFormula Report

Analysis Info

Analysis Name	D:\Data\USER-2013\MEI5022.d	Acquisition Date	7/24/2013 5:40:20 PM
Method	WU_tune_low_20121222.m	Operator	Ma
Sample Name	MEI5022	Instrument / Ser#	micrOTOF-Q II 10203
Comment			

Acquisition Parameter

Source Type	ESI	Ion Polarity	Positive	Set Nebulizer	0.3 Bar
Focus	Not active	Set Capillary	4500 V	Set Dry Heater	180 °C
Scan Begin	50 m/z	Set End Plate Offset	-500 V	Set Dry Gas	4.0 l/min
Scan End	3000 m/z	Set Collision Cell RF	150.0 Vpp	Set Divert Valve	Source

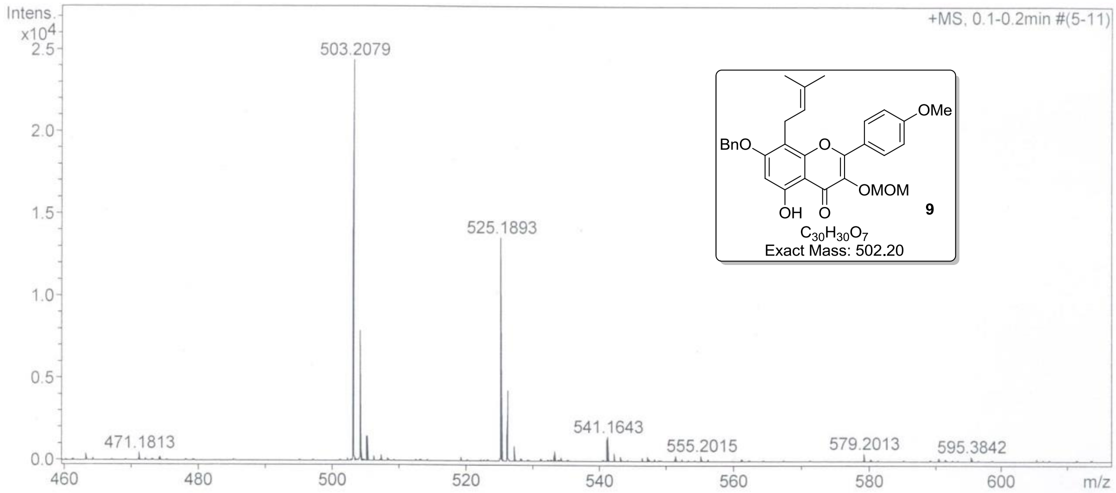

Meas. m/z	#	Formula	Score	m/z	err [ppm]	Mean err [ppm]	mSigma	rdb	e⁻ Conf	N-Rule
503.2079	1	C 30 H 31 O 7	100.00	503.2064	-3.0	-2.7	3.3	15.5	even	ok
525.1893	1	C 30 H 30 Na O 7	100.00	525.1884	-1.7	-1.6	6.2	15.5	even	ok

Bruker Compass DataAnalysis 4.0 printed: 7/24/2013 5:48:04 PM Page 1 of 1

Mass Spectrum SmartFormula Report

Analysis Info

Analysis Name	D:\Data\USER-2013\mei458.d	Acquisition Date	12/17/2013 6:34:30 PM
Method	WU_tune_low_20121222.m	Operator	Ma
Sample Name	mei458	Instrument / Ser#	micrOTOF-Q II 10203
Comment			

Acquisition Parameter

Source Type	ESI	Ion Polarity	Positive	Set Nebulizer	0.4 Bar
Focus	Not active	Set Capillary	4500 V	Set Dry Heater	180 °C
Scan Begin	50 m/z	Set End Plate Offset	-500 V	Set Dry Gas	4.0 l/min
Scan End	3000 m/z	Set Collision Cell RF	150.0 Vpp	Set Divert Valve	Source

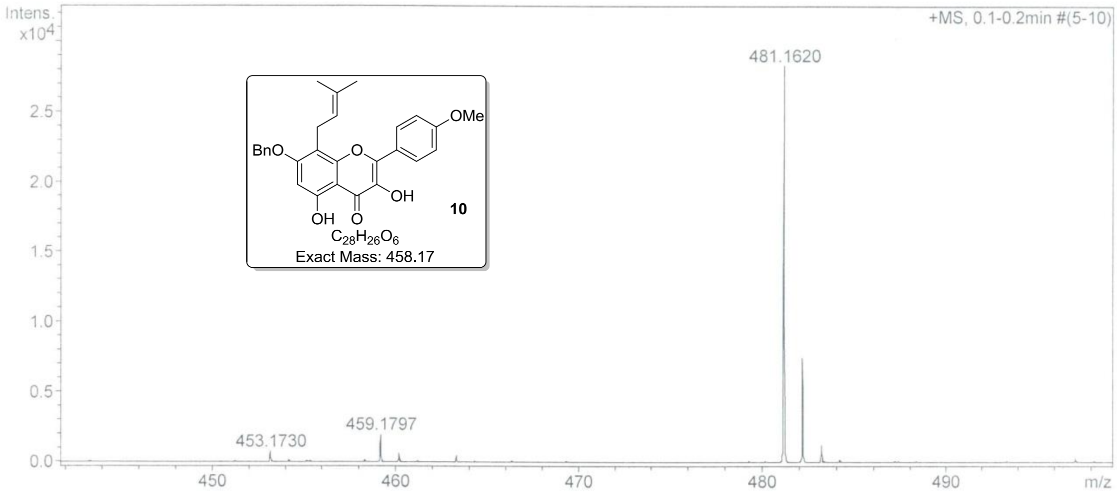

Meas. m/z	#	Formula	Score	m/z	err [ppm]	Mean err [ppm]	mSigma	rdb	e⁻ Conf	N-Rule
481.1620	1	C 28 H 26 Na O 6	100.00	481.1622	0.4	0.6	20.7	15.5	even	ok

Bruker Compass DataAnalysis 4.0 printed: 12/17/2013 6:39:42 PM Page 1 of 1

Mass Spectrum SmartFormula Report

Analysis Info

Analysis Name	D:\Data\USER-2013\MEI5021.d	Acquisition Date	7/24/2013 5:38:11 PM
Method	WU_tune_low_20121222.m	Operator	Ma
Sample Name	MEI5021	Instrument / Ser#	micrOTOF-Q II 10203
Comment			

Acquisition Parameter

Source Type	ESI	Ion Polarity	Positive	Set Nebulizer	0.3 Bar
Focus	Not active	Set Capillary	4500 V	Set Dry Heater	180 °C
Scan Begin	50 m/z	Set End Plate Offset	-500 V	Set Dry Gas	4.0 l/min
Scan End	3000 m/z	Set Collision Cell RF	150.0 Vpp	Set Divert Valve	Source

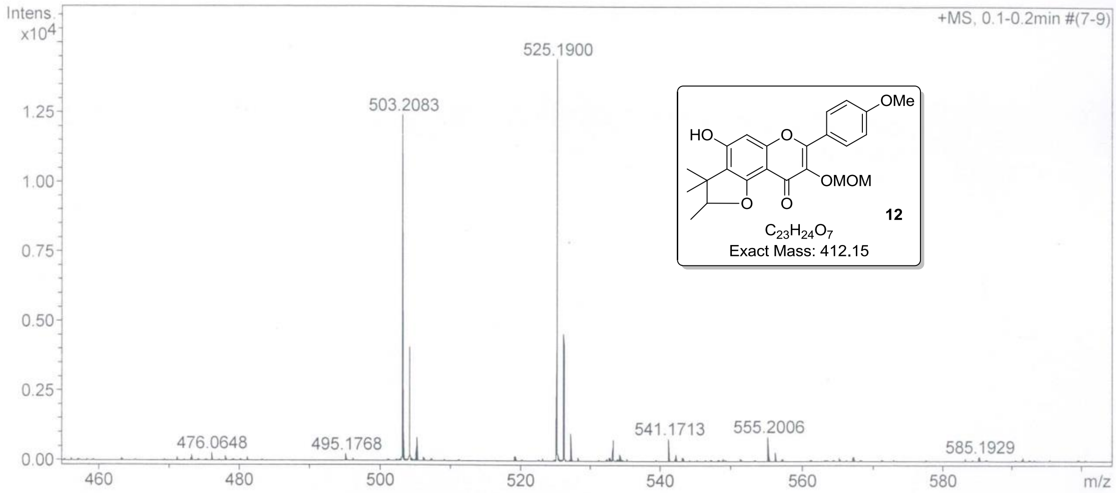

Meas. m/z	#	Formula	Score	m/z	err [ppm]	Mean err [ppm]	mSigma	rdb	e⁻ Conf	N-Rule
503.2083	1	C 30 H 31 O 7	100.00	503.2064	-3.7	-3.3	0.9	15.5	even	ok
525.1900	1	C 30 H 30 Na O 7	100.00	525.1884	-3.0	-2.6	4.0	15.5	even	ok

Bruker Compass DataAnalysis 4.0 printed: 7/24/2013 5:46:56 PM Page 1 of 1

Mass Spectrum SmartFormula Report

Analysis Info

Analysis Name	D:\Data\USER-2015\m970.d	Acquisition Date	2/4/2015 4:27:19 PM
Method	tune_wide.m	Operator	Ma
Sample Name	m970	Instrument / Ser#	micrOTOF-Q II 10203
Comment			

Acquisition Parameter

Source Type	ESI	Ion Polarity	Positive	Set Nebulizer	0.3 Bar
Focus	Active	Set Capillary	4500 V	Set Dry Heater	180 °C
Scan Begin	50 m/z	Set End Plate Offset	-500 V	Set Dry Gas	4.0 l/min
Scan End	3000 m/z	Set Collision Cell RF	650.0 Vpp	Set Divert Valve	Source

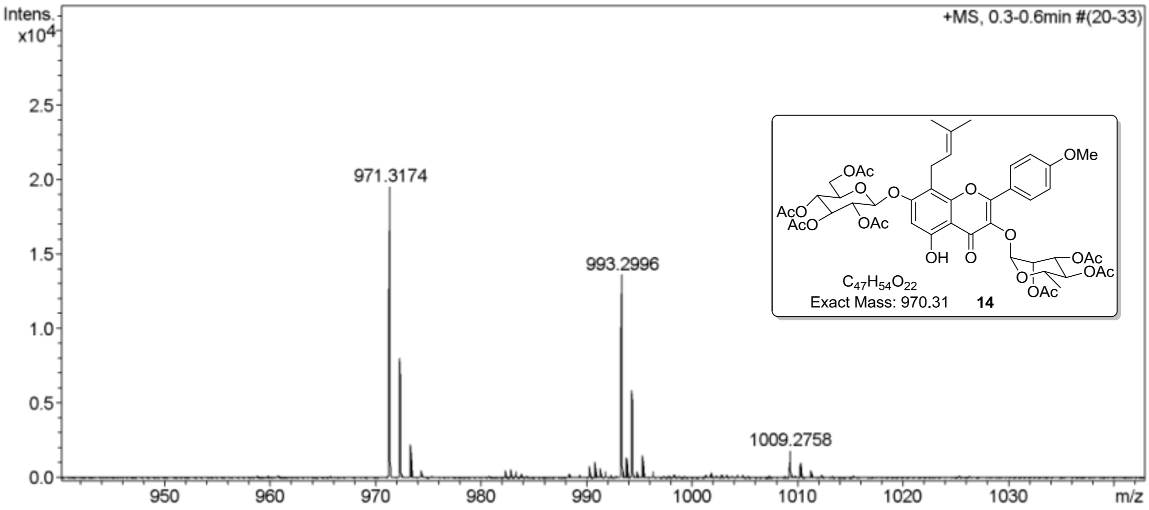

Meas. m/z	#	Formula	Score	m/z	err [ppm]	Mean err [ppm]	mSigma	rdb	e⁻ Conf	N-Rule
971.3174	1	C 47 H 55 O 22	100.00	971.3179	0.6	0.1	59.6	20.5	even	ok
993.2996	1	C 47 H 54 Na O 22	100.00	993.2999	0.3	-0.4	52.6	20.5	even	ok
1009.2758	1	C 47 H 54 K O 22	100.00	1009.2738	-2.0	-1.4	38.5	20.5	even	ok

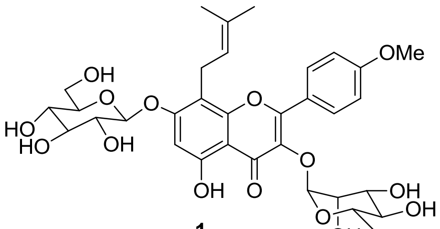

HMBC Spectrum of Compound **1**

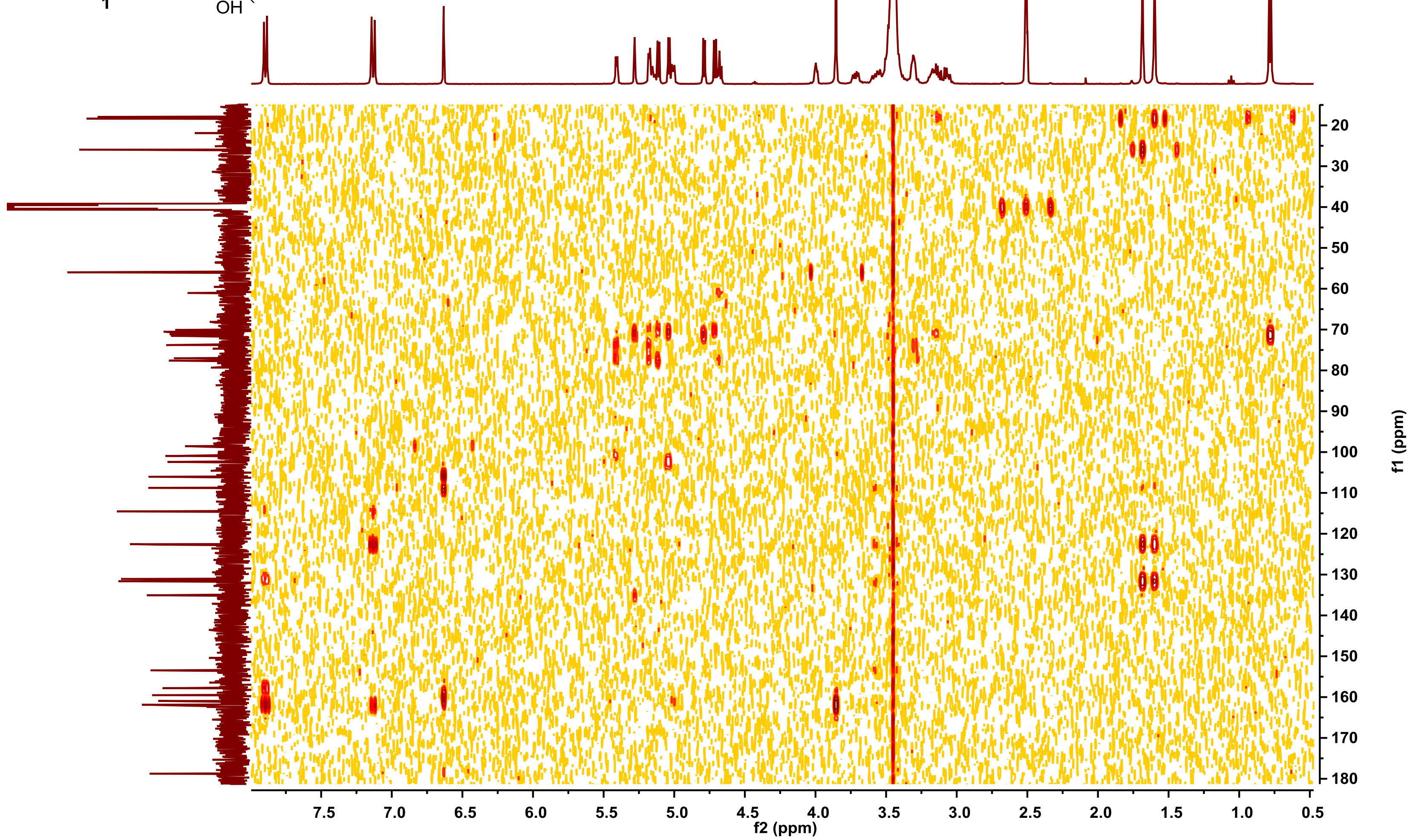

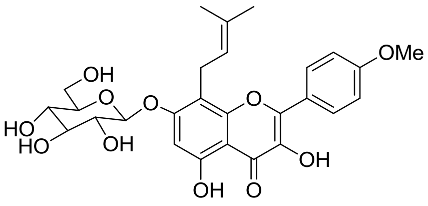

2

HMBC Spectrum of Compound **2**

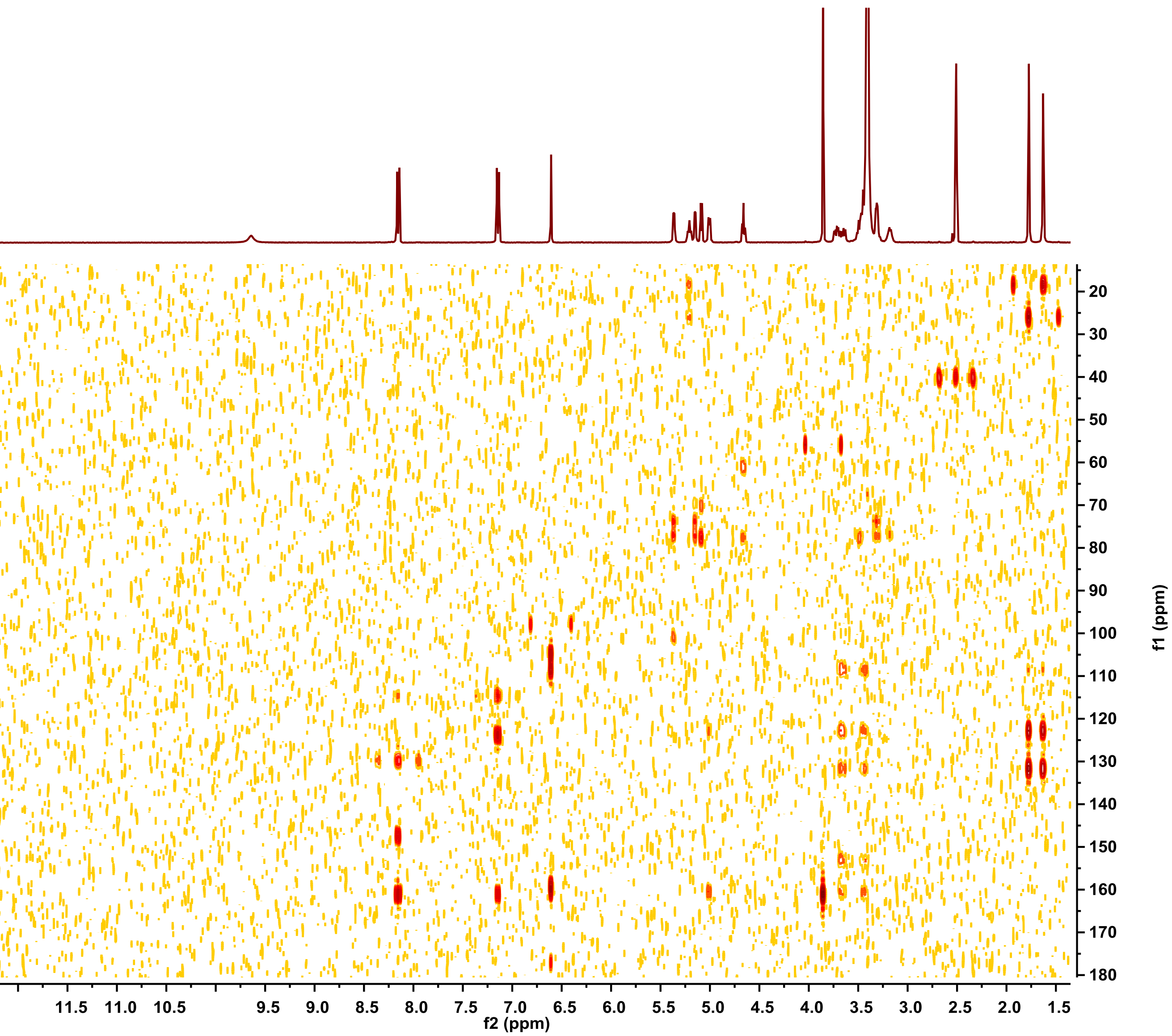

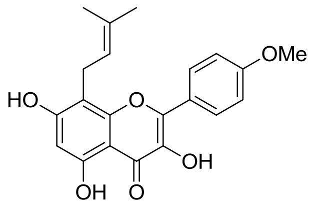

3

HMBC Spectrum of Compound **3**

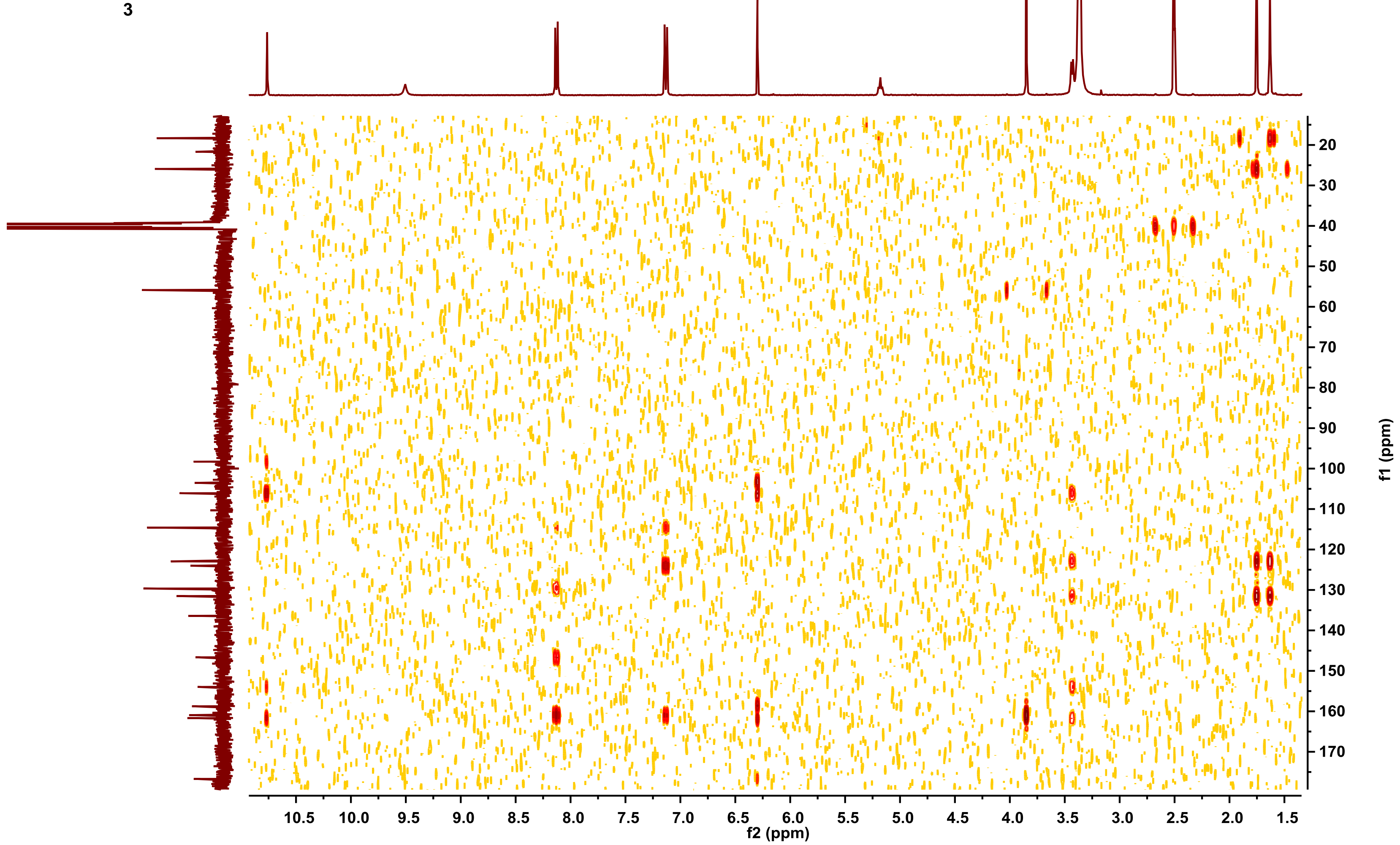

HMBC Spectrum of Compound **7**

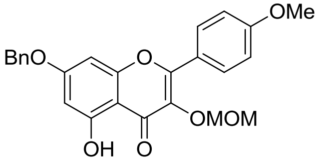

7

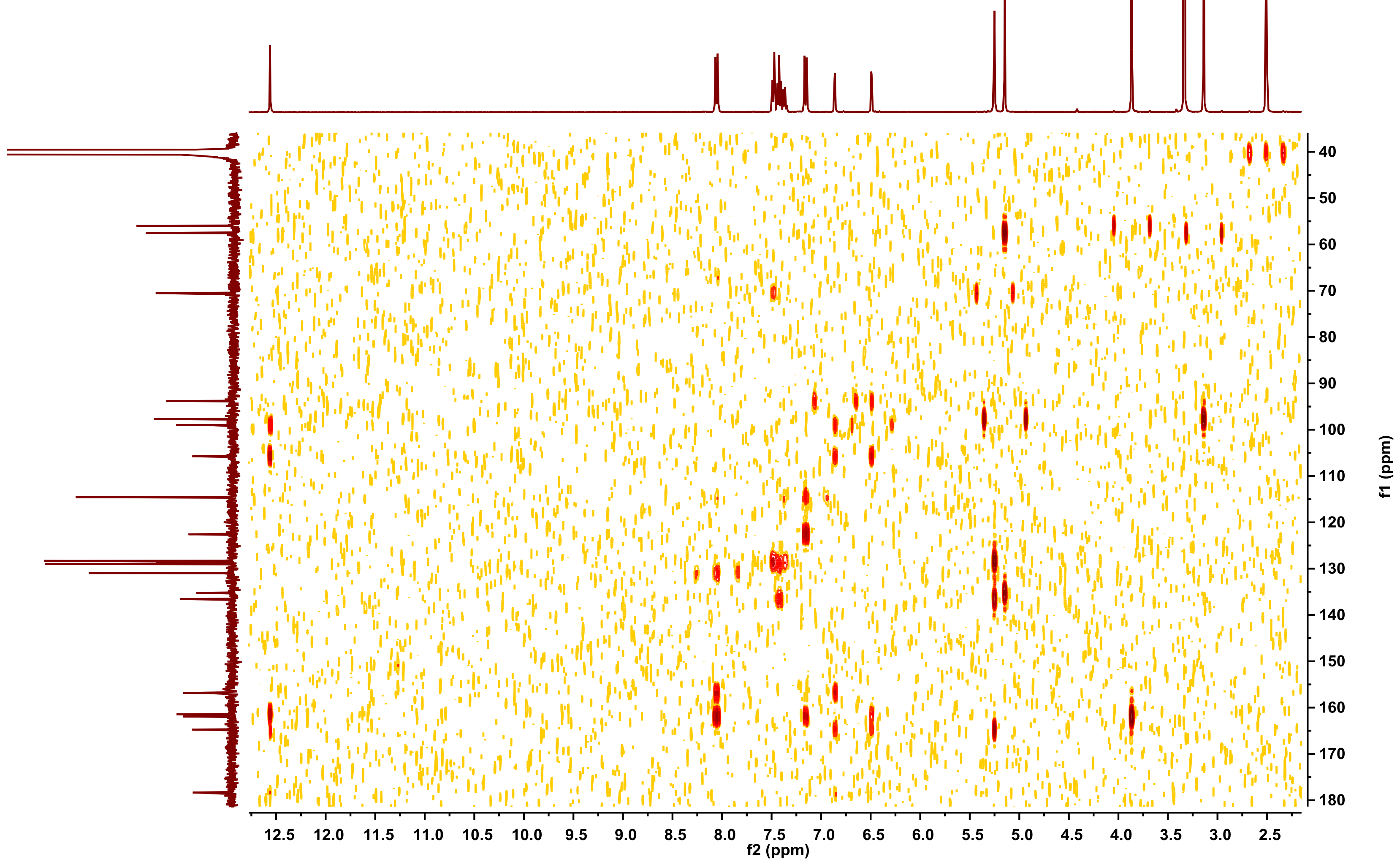

HMBC Spectrum of Compound **8**

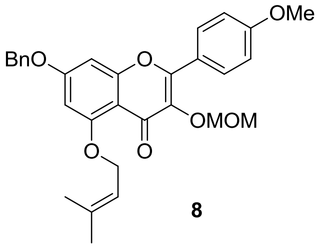

8

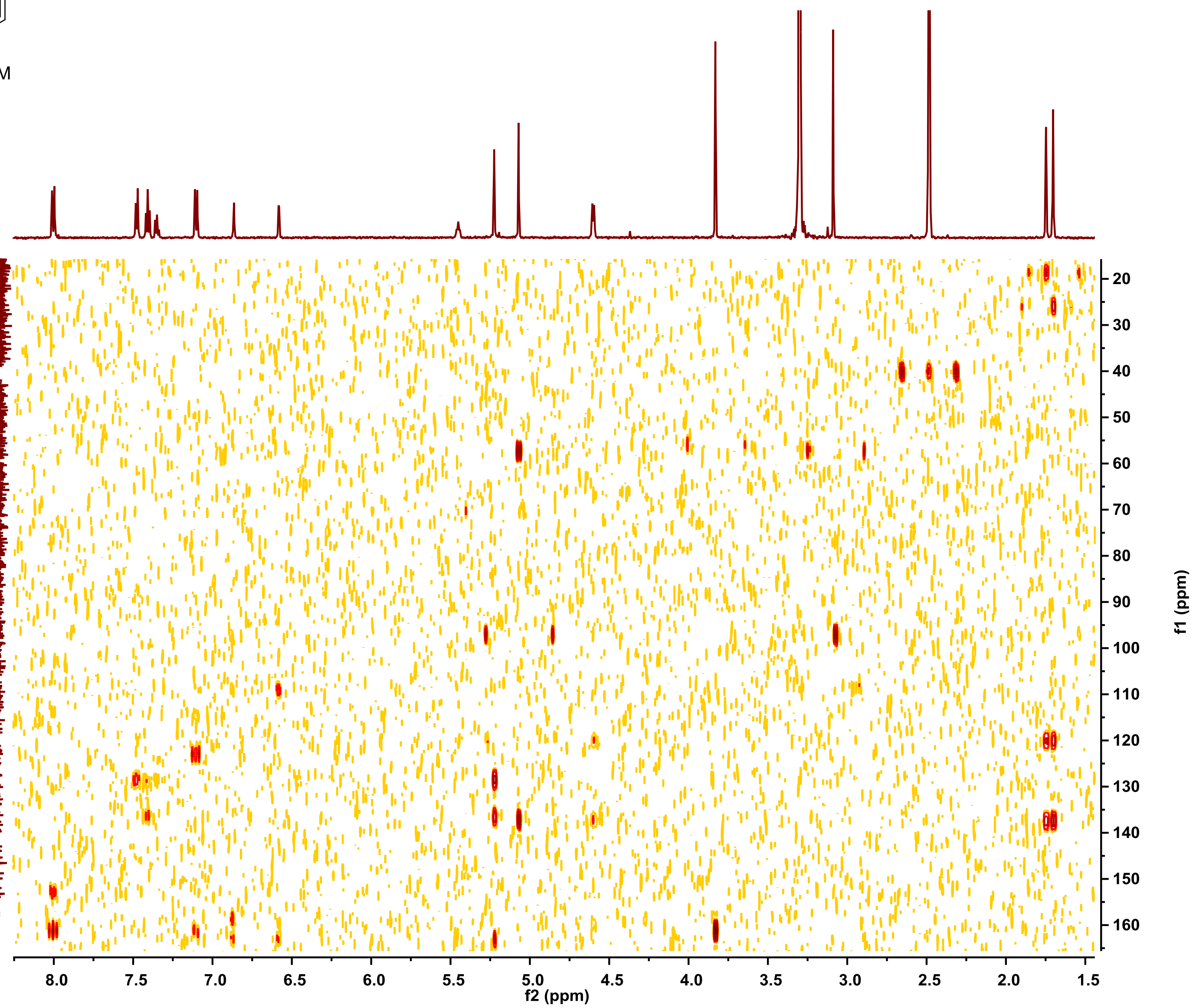

HMBC Spectrum of Compound **9**

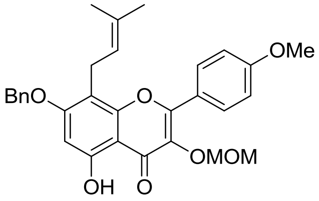

9

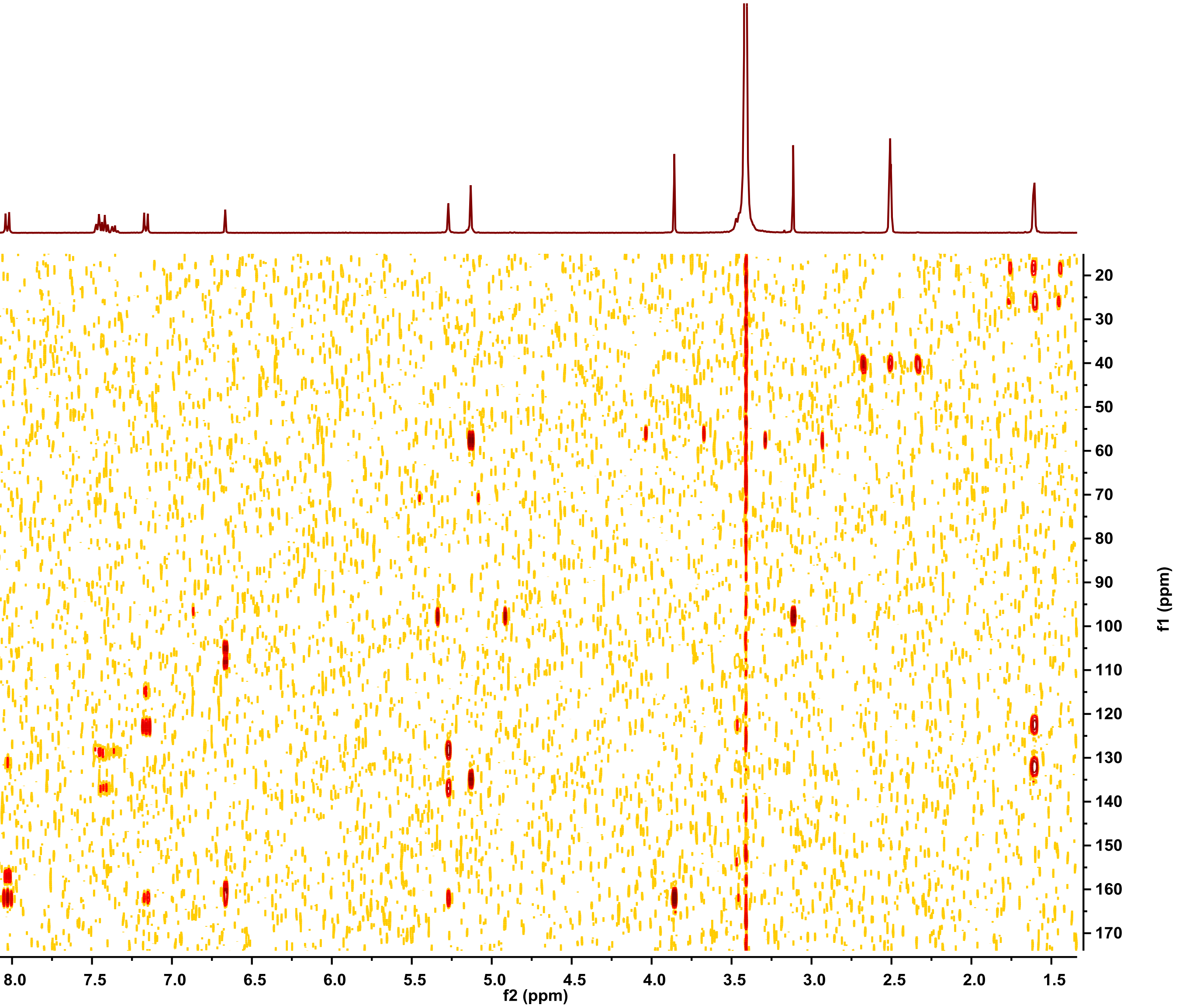

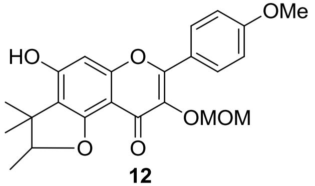

12

HMBC Spectrum of Compound **12**

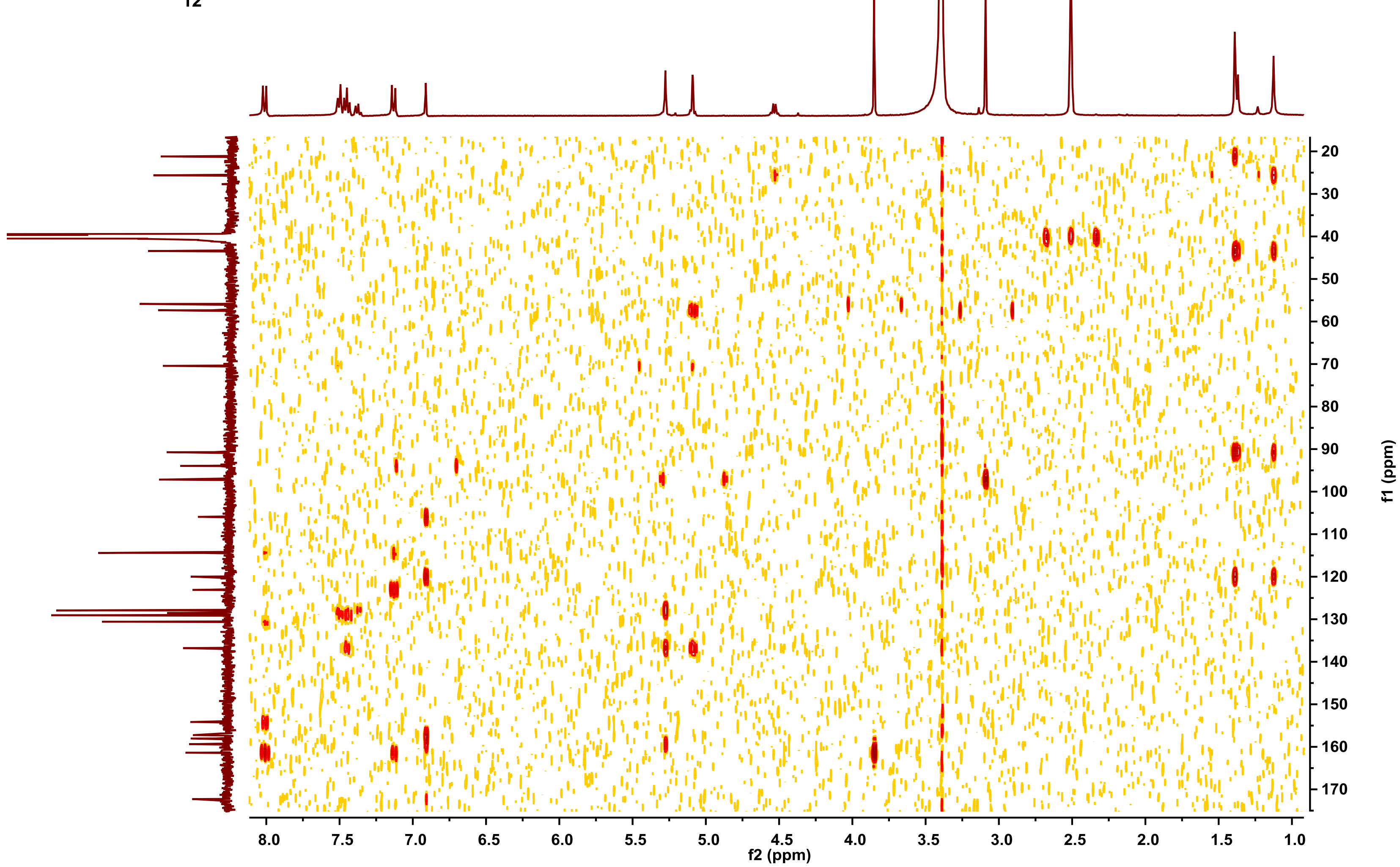

HMBC Spectrum of Compound **14**

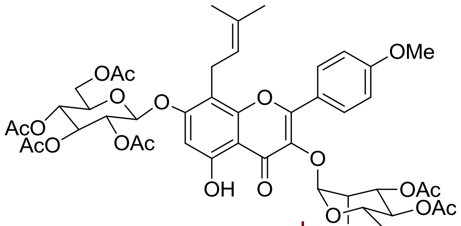

14

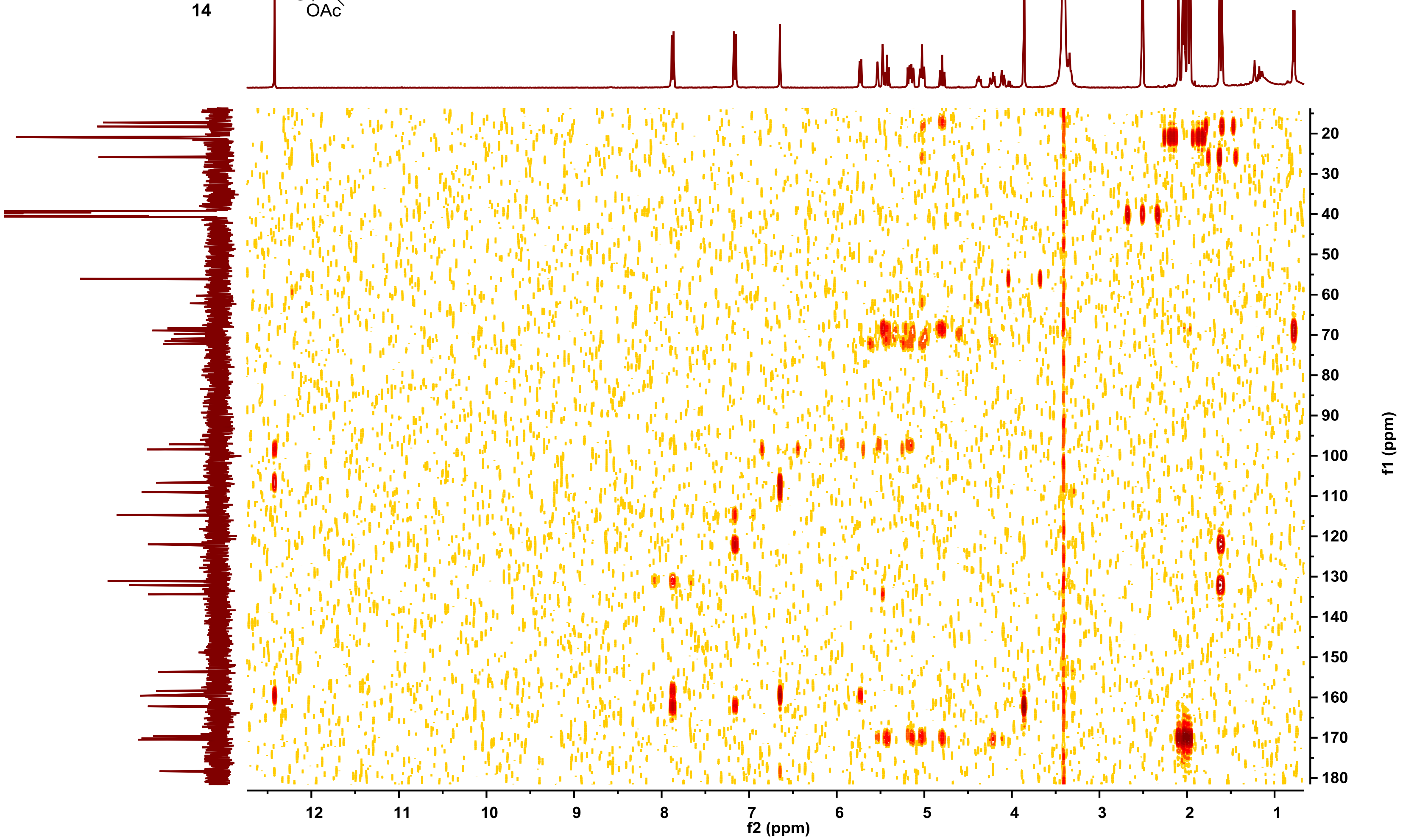

HSQC Spectrum of Compound **1**

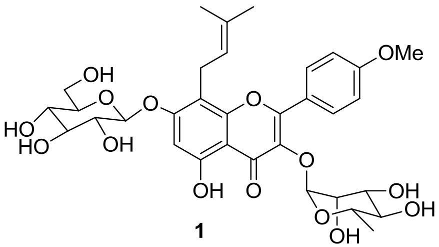

1

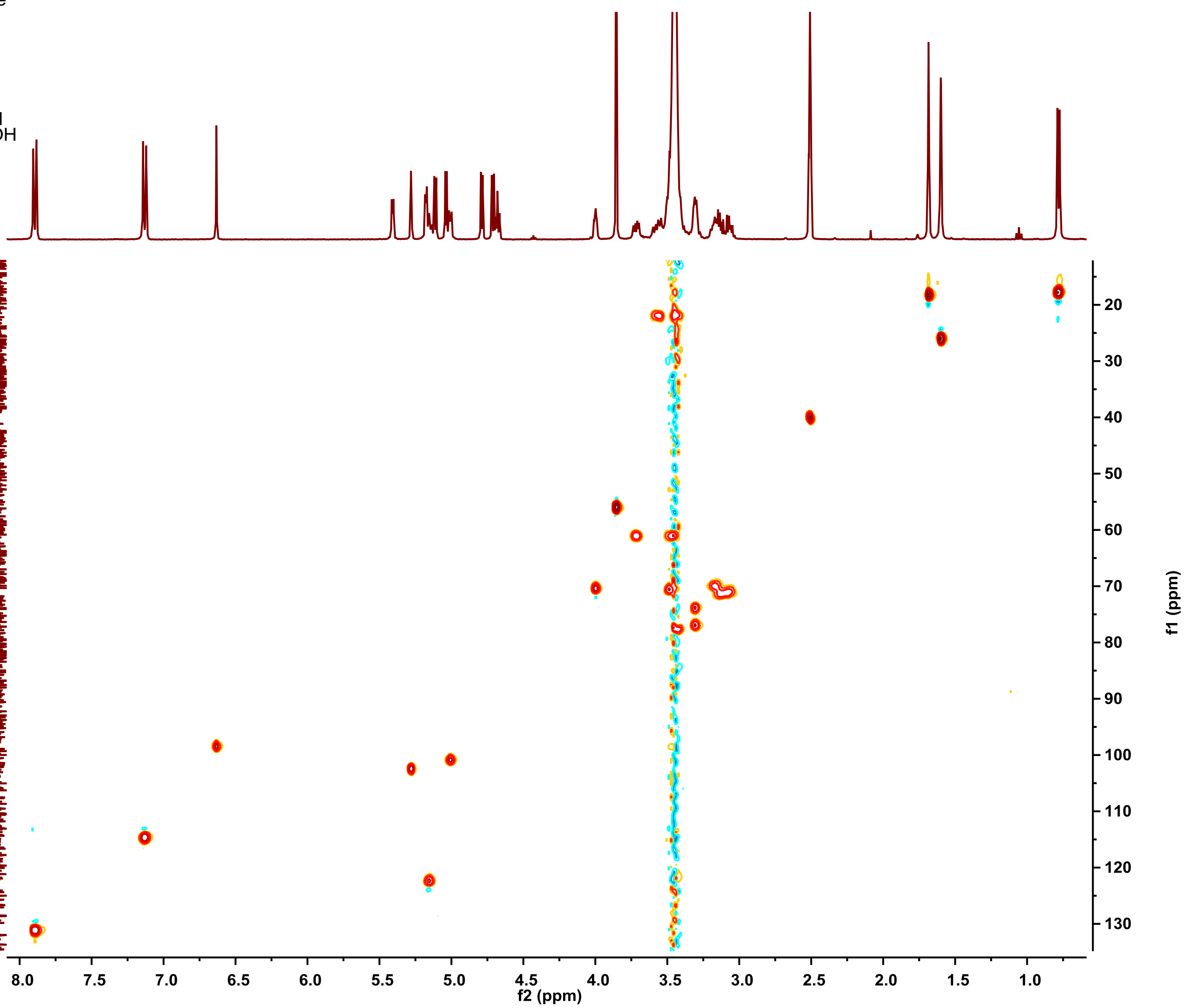

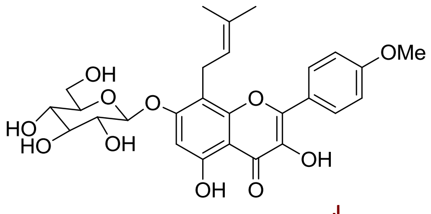

2

HSQC Spectrum of Compound **2**

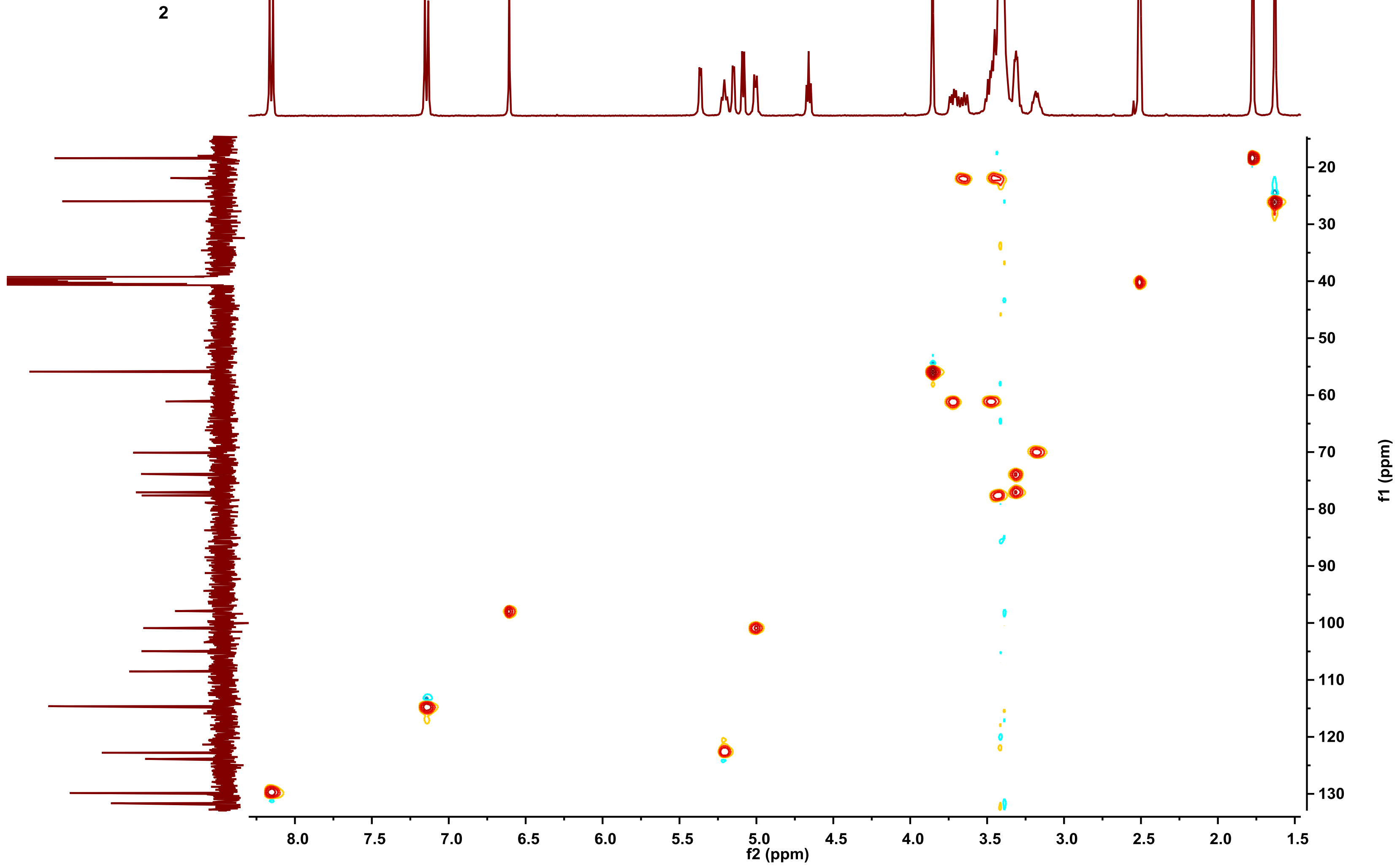

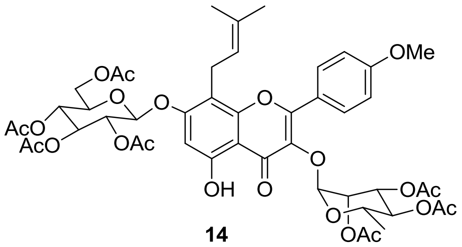

14

HSQC Spectrum of Compound **14**

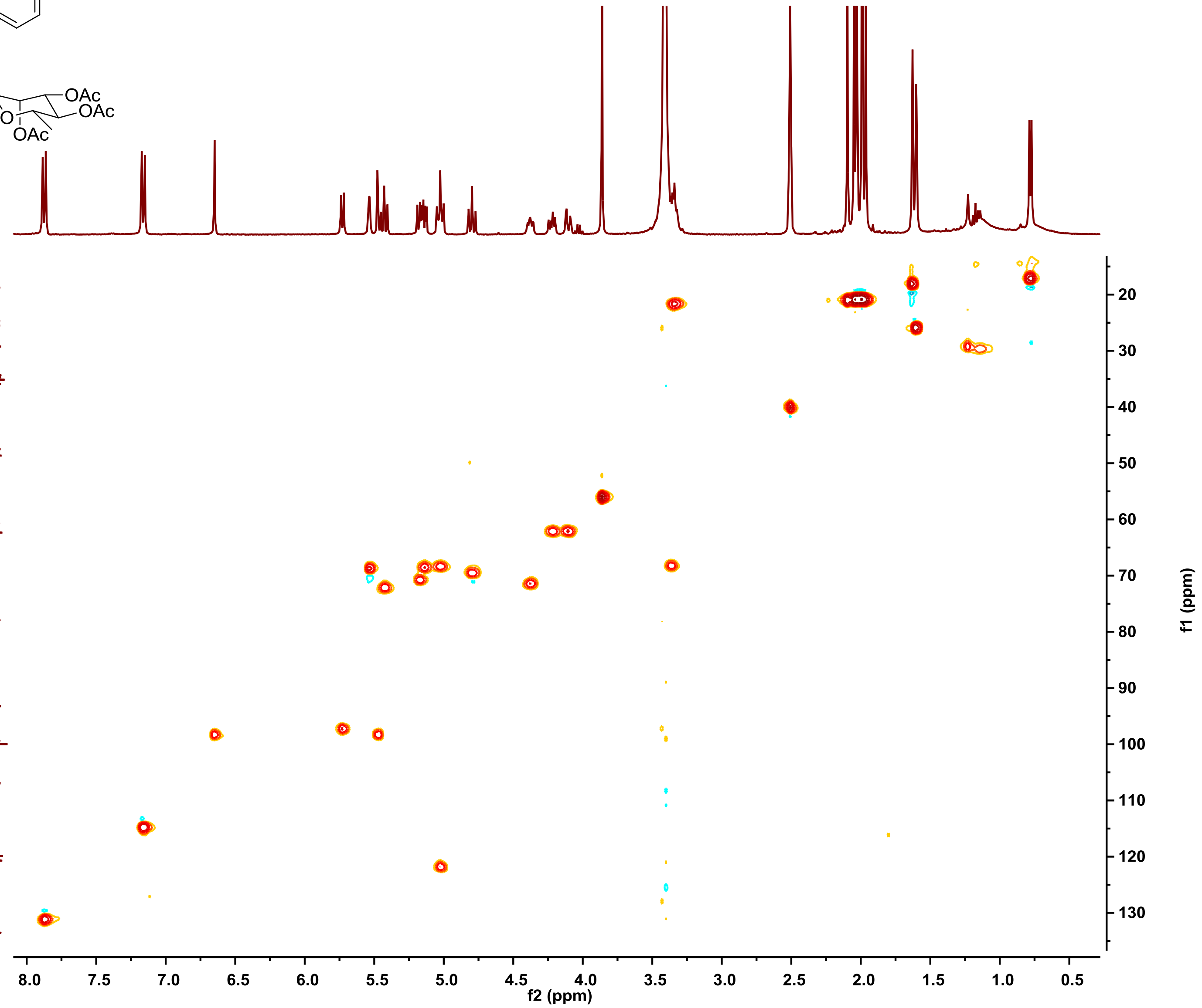

COSY Spectrum of Compound **1**

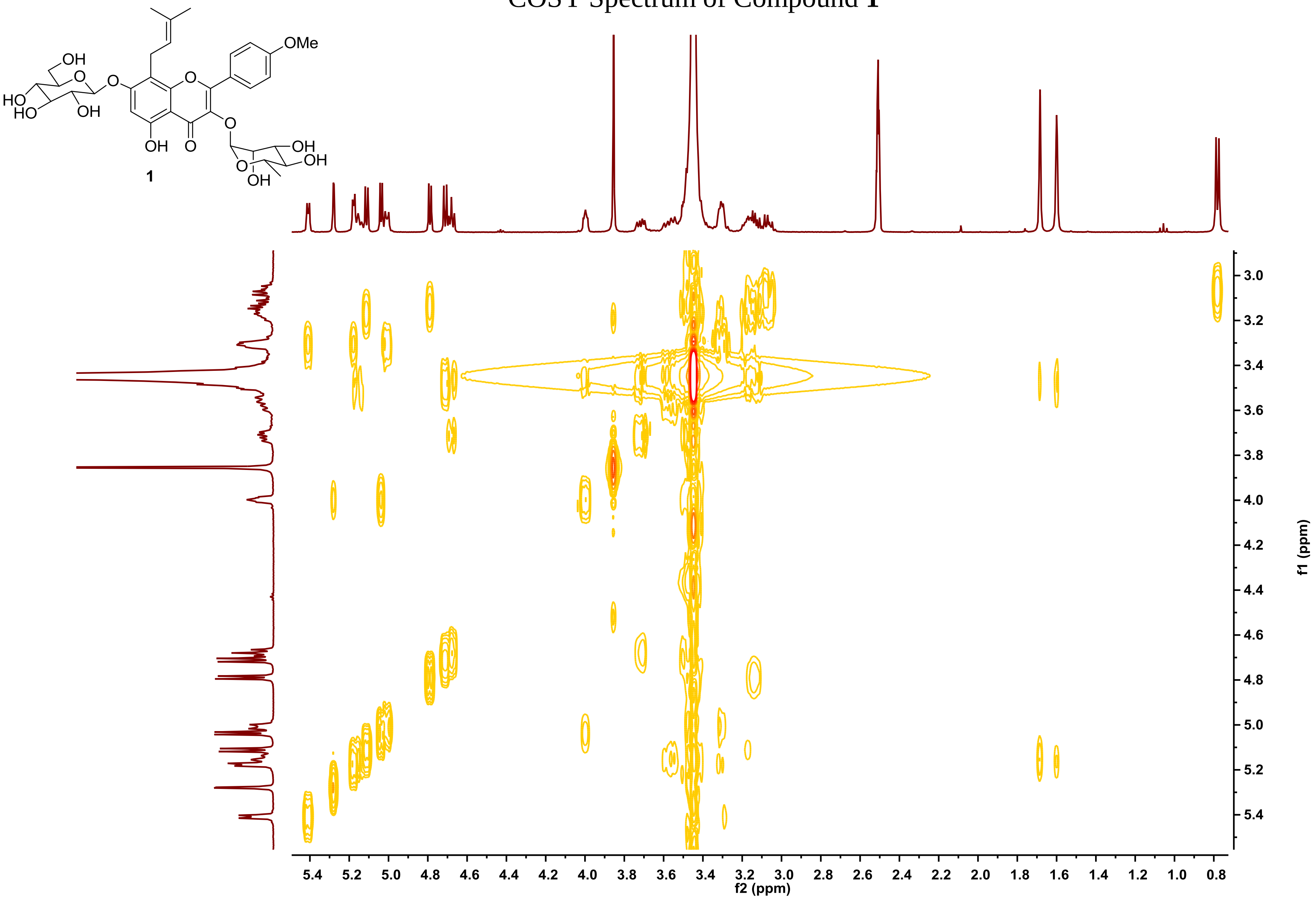

1

COSY Spectrum of Compound **2**

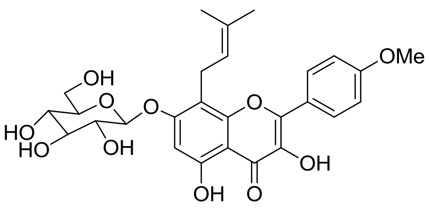

2

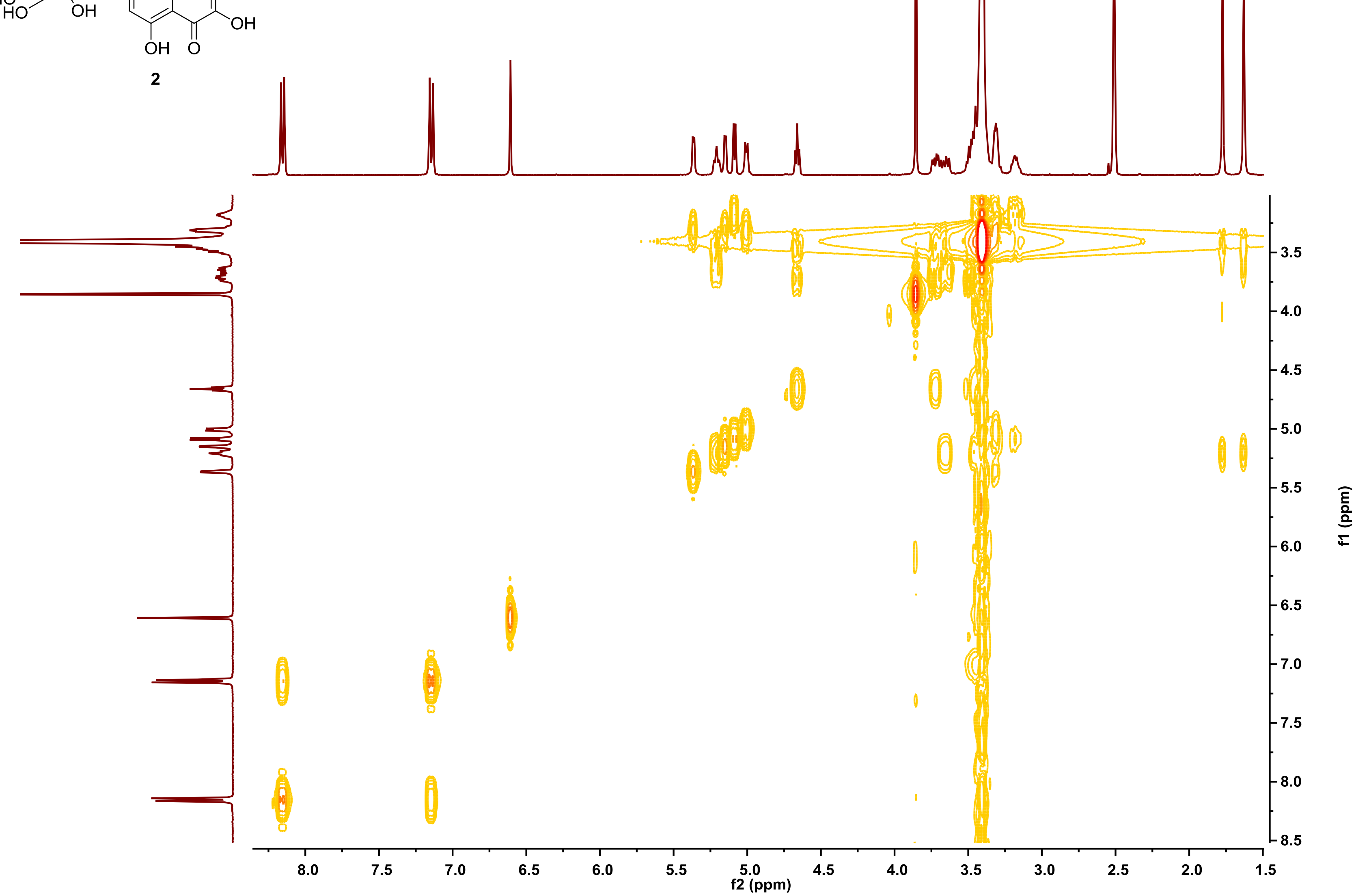

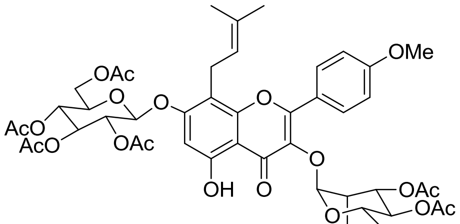

14

COSY Spectrum of Compound **14**

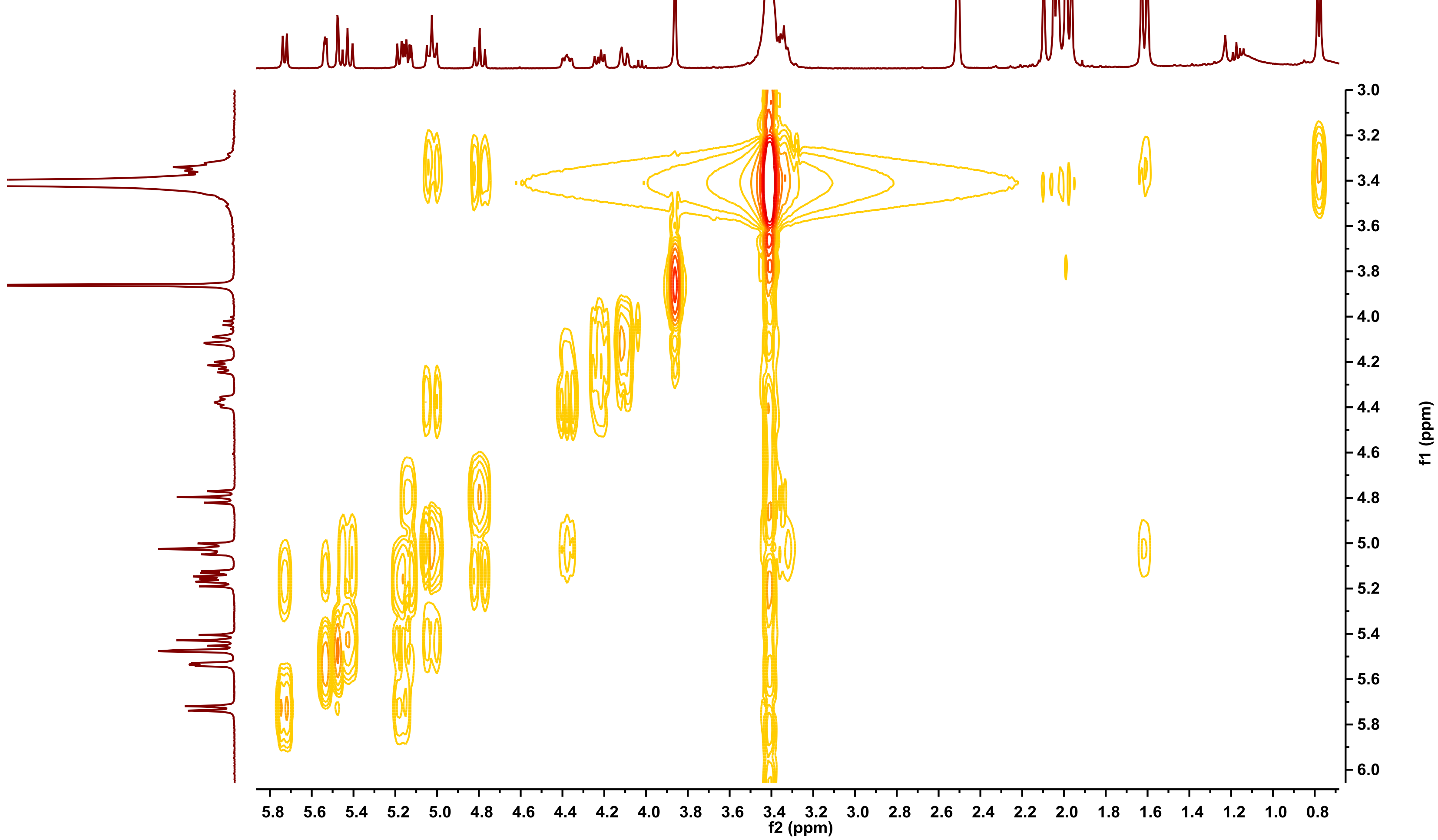

NOESY Spectrum of Compound **3**

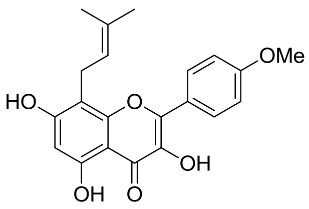

3

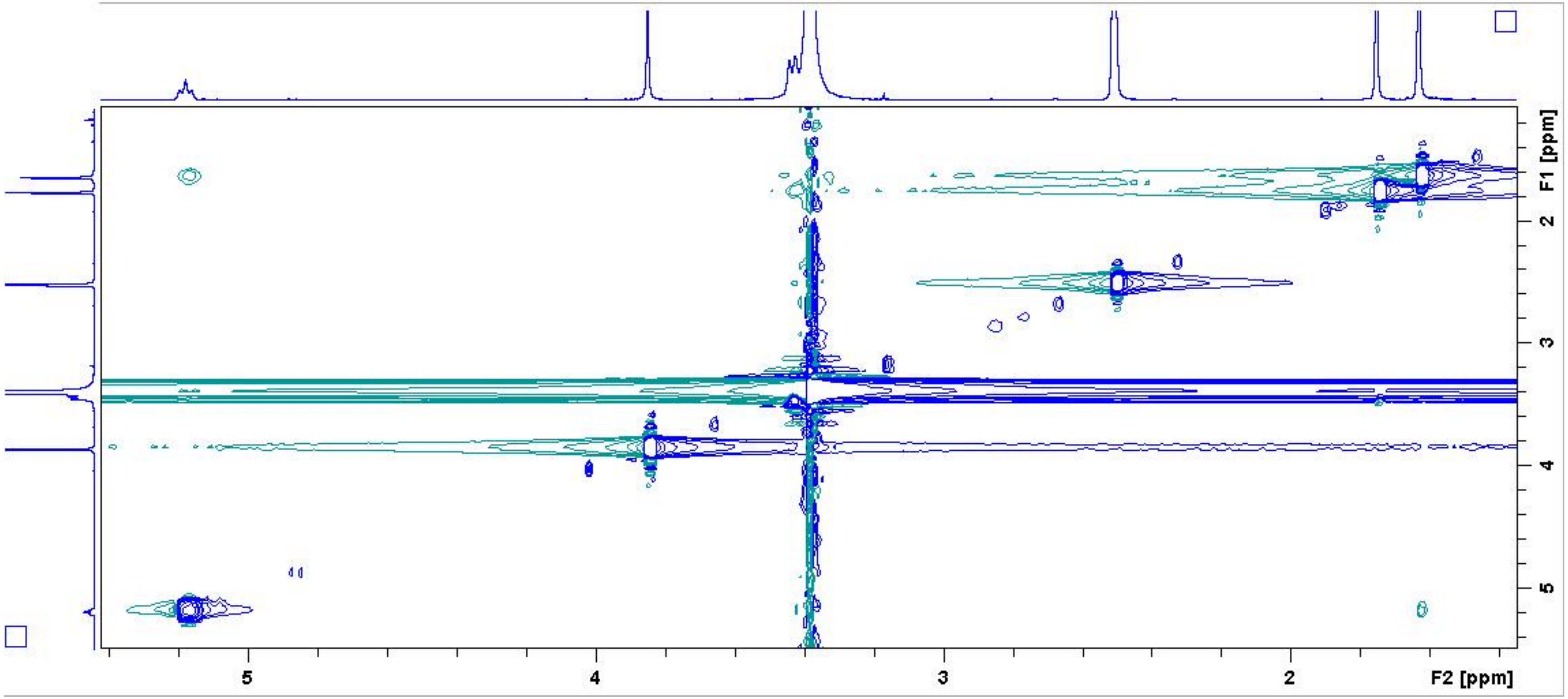

Supplement: File 1 — Experimental section and copies of NMR, ESI-HRMS, HMBC, HSQC, COSY and NOESY spectra. [file Beilstein_J_Org_Chem-11-1220-s001.pdf]
